# Mass Spectrometry in Allogeneic CAR-T Cell Manufacturing: From Cellular Starting Materials to Multi-Attribute Quality Control

**DOI:** 10.3390/cells15151333

**Published:** 2026-07-25

**Authors:** Naryeong Kim, Zhouyang Huang, Michael Born, Julien Camperi

**Affiliations:** 1 Cell Therapy Engineering and Development, Genentech South San Francisco, 1 DNA Way, South San Francisco, CA 94080, USAhuang.tracky@gene.com (Z.H.); 2 Cell Therapy Engineering and Development, Genentech San Diego, 9390 Towne Centre Dr., San Diego, CA 92121, USA

**Keywords:** allogeneic CAR T-cell therapy, mass spectrometry, proteomics, metabolomics, cell therapy manufacturing, quality control, biomarker analysis

## Abstract

Chimeric antigen receptor (CAR) T-cell therapies have demonstrated remarkable clinical efficacy in hematological malignancies, yet their broader application is constrained by manufacturing complexity and variability, particularly in autologous settings. The development of allogeneic CAR T-cell therapies offers a promising alternative by enabling scalable, “off-the-shelf” production; however, these approaches introduce additional challenges related to donor variability, genome editing, and product consistency. Robust analytical strategies are therefore required to ensure safety, efficacy, and batch-to-batch reproducibility. Conventional analytical methods, such as flow cytometry and enzyme-linked immunosorbent assays, provide targeted, high-confidence measurements of predefined cellular and soluble markers but are inherently limited in their ability to capture the full molecular and functional complexity of CAR T-cell products. In current manufacturing paradigms, these assays are typically deployed as isolated quality control readouts rather than as components of an integrated control strategy that links donor variability, gene-editing material quality, in-process metabolic state, final product critical quality attributes, and clinical biomarker responses. In this context, mass spectrometry (MS) has emerged as a powerful platform for high-dimensional molecular characterization, enabling analysis of gene-editing reagents, proteins, metabolites, lipids, and both culture and spent-media composition across the CAR T-cell manufacturing workflow. In this review, we examine the various applications and tools of MS across key stages of the allogeneic CAR T-cell workflow, including donor characterization, analysis of gene-editing materials, in-process culture monitoring, drug product quality assessment, and post-infusion biomarker evaluation. Collectively, these approaches demonstrate the potential of MS-driven analytics to address current limitations in CAR T-cell manufacturing by improving process understanding, enabling comprehensive quality assessment, and supporting regulatory decision-making. The integration of MS into CAR T-cell workflows may ultimately facilitate the development of more consistent, scalable, and effective cell therapies.

## 1. Introduction

Chimeric antigen receptor (CAR) T-cell therapies have transformed the treatment landscape for hematological malignancies. In 2017, the U.S. Food and Drug Administration (FDA) approved the first CAR T-cell products, tisagenlecleucel (Kymriah) and axicabtagene ciloleucel (Yescarta), for the treatment of acute lymphoblastic leukemia and large B-cell lymphoma, respectively [1,2]. These approvals demonstrated the remarkable efficacy of CAR T-cell therapies in patients with relapsed or refractory disease [3]. However, early-generation CAR T-cell products are predominantly autologous, requiring the use of a patient’s own cells. This individualized approach presents significant logistical and manufacturing challenges, including variable starting material quality, complex supply chains, and prolonged production timelines.

To address these limitations, allogeneic CAR T-cell therapies are being developed using healthy donor-derived cells to generate “off-the-shelf” products. This strategy enables large-scale manufacturing, facilitates cell banking, and has the potential to reduce costs while improving treatment accessibility [4,5,6]. Nevertheless, allogeneic approaches introduce additional biological and technical challenges. Donor-derived T cells must be engineered to prevent graft-versus-host disease (GvHD) and to evade host immune rejection, typically through targeted modification of T-cell receptors and other immune-related genes [7,8,9]. These genome-editing steps increase process complexity and necessitate rigorous analytical evaluation, not only to ensure editing precision and detect unintended genomic alterations [10,11,12], but also to characterize gene-editing components, including guide RNAs (gRNAs), CRISPR-associated protein 9 (Cas9), DNA plasmids, and viral vectors, before their use in the manufacturing process. Accordingly, regulatory agencies such as the FDA require comprehensive safety and quality assessments for genome-edited cell therapies [11].

The manufacturing of allogeneic CAR T-cell products involves multiple stages, including donor cell selection and activation, genetic modification, expansion, formulation, and cryopreservation, each of which can introduce variability in cellular phenotype, function, and product composition. Ensuring batch-to-batch consistency therefore remains a major analytical challenge [4,5,6,7]. The primary analytical methods used in CAR-T manufacturing include flow cytometry, enzyme-linked immunosorbent assays (ELISA), qPCR/ddPCR-based vector copy number analysis, sterility and mycoplasma testing, endotoxin testing, viability assays, and cell-based potency assays. While these methods remain foundational for release and safety testing, they remain analyte-specific, end-point-based, or limited in molecular breadth [13,14,15]. Mass spectrometry (MS) therefore offers a complementary approach for measuring proteins, peptides, metabolites, lipids, and process-related impurities [16,17,18,19], providing a powerful tool for a more comprehensive view of cellular state and product quality [17,18].

In parallel, the regulatory landscape for advanced cell therapies is evolving rapidly, with increasing emphasis on analytical rigor, process understanding, and control strategies [20]. Frameworks such as quality by design (QbD) and process analytical technology (PAT) [21] highlight the need for comprehensive characterization of critical quality attributes (CQAs) and their impact on clinical performance. However, defining and monitoring CQAs for complex, living cell products such as allogeneic CAR T cells remains particularly challenging due to their inherent heterogeneity and dynamic nature [22]. This has created a growing demand for high-dimensional, systems-level analytical approaches capable of capturing molecular complexity and supporting robust comparability, release testing, and lifecycle management. In this context, MS is best viewed as an orthogonal layer that can support CQA identification, targeted method development, impurity characterization, metabolic process monitoring, and comparability assessment.

Despite its growing application in CAR T-cell research, the use of mass spectrometry remains fragmented. Existing studies typically address discrete analytical questions rather than establishing an integrated framework that spans the full lifecycle of allogeneic CAR T-cell manufacturing. As a result, the potential of MS to systematically characterize variability in critical materials, define and link critical material attributes (CMAs) to CQAs, and support regulatory decision-making across the manufacturing continuum has not yet been fully realized. In this review, we examine the application of MS across the allogeneic CAR T-cell manufacturing workflow. We emphasize liquid chromatography–mass spectrometry (LC-MS) approaches for proteomic, metabolomic, lipidomic, and impurity analysis, while also discussing complementary modalities such as matrix-assisted laser desorption/ionization (MALDI-MS), gas chromatography–mass spectrometry (GC–MS), and MS-enabled single-cell technologies where relevant [17,18]. We organize these applications by workflow stage, including donor characterization, gene-editing material assessment, in-process culture monitoring, final drug product characterization, and post-infusion biomarker evaluation, ultimately providing insight into how MS brings improved process understanding and contributes to the development of consistent, safe, and effective allogeneic CAR T-cell therapies [4,5,6,7,17,18]. Although this review focuses on allogeneic workflows, many of the MS-driven analytical modalities detailed herein—including gene editing characterization, process impurity tracking, and multi-attribute product profiling—provide a universal framework that extends to autologous CAR-T, TCR-T, CAR-NK platforms, and other immune cell types.

## 2. From Donor to Drug Product: Multi-Stage Manufacturing of Allogeneic CAR-T Cells

**Figure 1:**
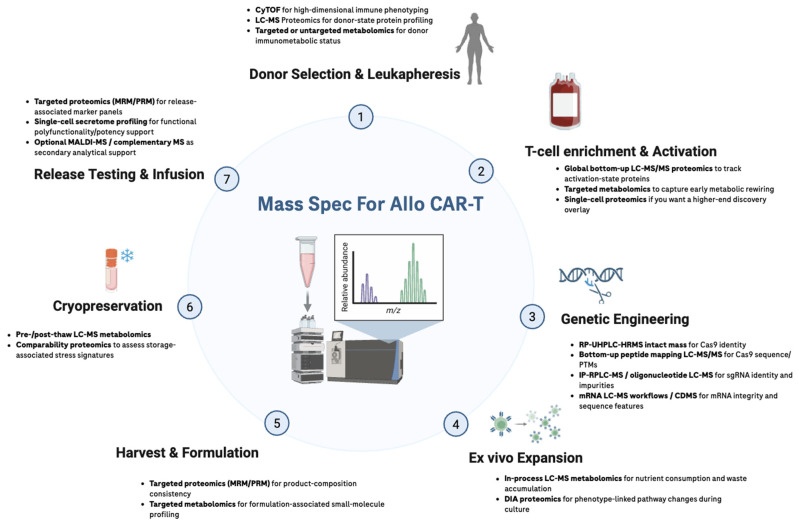
Allogeneic CAR-T cell manufacturing workflow from leukapheresis to patient reinfusion, highlighting stages where mass spectrometry applications can support process monitoring and product characterization.

Allogeneic CAR-T cell manufacturing is a multi-stage, tightly controlled process designed to generate a uniform, cryopreserved cell therapy product from healthy donor starting material for administration to multiple patients. Compared with autologous manufacturing, which is individualized and patient-specific, allogeneic workflows must achieve lot-level consistency, scalability, and reproducibility across expanded production runs [5,20,23,24,25]. The process typically begins with donor selection and leukapheresis. Following this collection, donor T-cells are enriched and activated using antibody-based stimulation to promote proliferation and render the cells permissive to genetic modification [4,20]. Subsequent steps include genetic engineering, ex vivo expansion, harvest and formulation, cryopreservation, and final product release testing [20,23,26] (Figure 1).

To mitigate the inherent variability of these unit operations and safeguard the identity, purity, potency, and safety of the final drug product, robust analytical characterization must be integrated throughout manufacturing [24,26,27,28]. Specifically, phase-appropriate analytics are required to confirm the biological suitability of the starting material, the structural consistency of the gene-editing reagents, the metabolic and phenotypic stability of the expanding culture, and the critical molecular features of the final cryopreserved product [20,25,29].

Following activation, genetic engineering typically includes two related but distinct steps: first, the introduction of the CAR transgene; and second, for allogeneic products, additional genome modifications to reduce alloreactivity or improve persistence. CAR expression can be achieved using viral vectors (γ-retroviral or lentiviral), nonviral transposon systems, mRNA delivery, or targeted knock-in approaches, whereas allogeneic-enabling edits are commonly introduced using CRISPR/Cas9, TALENs, zinc-finger nucleases, or other nuclease-based systems [30,31,32]. In allogeneic CAR-T products, endogenous T-cell receptor components (e.g., TRAC) are frequently disrupted to prevent graft-versus-host disease, and additional edits like β2-microglobulin (B2M) knockout or immune-evasive transgene insertion are used to mitigate host immune rejection [30,33]. These multiplex editing strategies increase manufacturing complexity and introduce regulatory considerations regarding off-target activity, chromosomal rearrangements, and residual editing reagents [32,34]. Each additional edit involves the use of nucleic acid, protein, or delivery-component inputs whose identity, purity, degradation products, sequence-related impurities, and residual carryover can affect editing consistency or final-product safety, thereby expanding the analytical burden [10].

Next, the modified CAR-T cells undergo controlled ex vivo expansion in bioreactors or closed culture systems under defined cytokine and media conditions [23,25]. Expansion kinetics, metabolic state, and differentiation trajectory during this phase are closely linked to final product potency and persistence [35,36]. Because expansion is a dynamic biological selection process, variables such as nutrient availability, waste accumulation, oxygen transfer, cytokine dosing, and culture duration can shift the balance among proliferative, memory-like, effector, exhausted, and stressed cellular states [35,36,37]. Given the dynamic and living nature of the product, this stage requires a quality-by-design approach where in-process monitoring controls variability rather than relying solely on end-point testing [24,25,26,27,28,36]. Here, MS-based metabolomic, proteomic, lipidomic, and secretome measurements can not only support process understanding by linking culture conditions to molecular product state, but also provide actionable, near-term readouts for feeding strategy, media exchanges, process-drift investigations, and harvest-timing decisions [18,29].

After expansion, CAR-T cells are harvested, formulated with cryoprotectants (commonly dimethyl sulfoxide), and cryopreserved for storage and distribution [23,38]. Cryopreservation is a major product-quality stressor, because freezing, storage, shipment, and thawing can affect membrane integrity, mitochondrial function, oxidative stress, apoptotic priming, and post-thaw recovery [20,38,39]. Because each allogeneic CAR-T batch serves multiple patients, regulatory agencies expect comprehensive characterization to mitigate patient risk [20,34]. Prior to release, products undergo stringent quality control testing to confirm CAR expression, vector copy number, functional cytotoxic activity, sterility, and the absence of residual TCR-positive cells, mycoplasma, and endotoxin [20,26,27]. However, these standard release assays are typically targeted and endpoint-based; they do not necessarily resolve the broader proteomic, metabolic, lipidomic, or stress-response states that may influence comparability, stability, or post-thaw performance. This motivates deeper molecular characterization during formulation development, stability assessment, comparability studies, and failed-lot investigations [17,19,20].

As manufacturing platforms evolve toward increased genetic complexity and scale, advanced analytical tools, including mass spectrometry, are increasingly being explored to complement traditional assays and strengthen process understanding across the full manufacturing lifecycle [17,24]. MS is best viewed as a stage-specific, orthogonal analytical layer rather than a single universal assay:

- **Donor-stage:** Captures baseline molecular variability to inform downstream outcomes.
- **Gene-editing material:** Monitors and ensures the high quality of critical reagent components.
- **In-process:** Reveals metabolic or phenotypic drift during expansion.
- **Drug product:** Supports CQA discovery/identification, comparability, impurity investigation, and post-thaw quality assessment.

**Figure 2:**
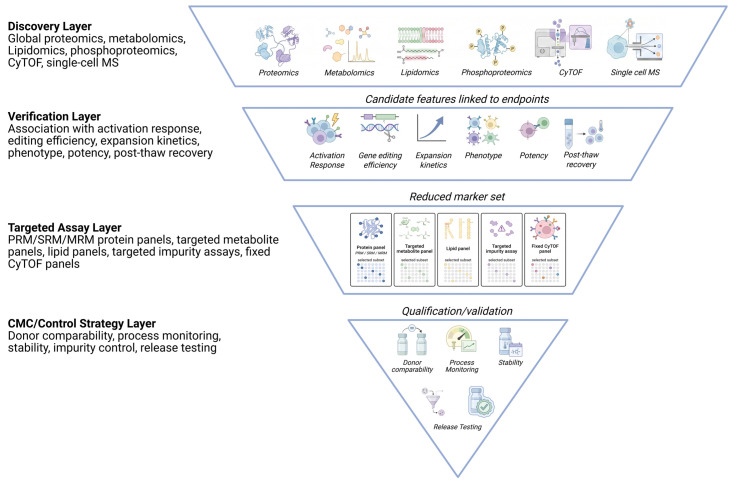
**MS Translation Ladder: From Discovery Omics to Actionable CQA/Control Assays.** The diagram illustrates the progressive funneling of mass spectrometry application from discovery-based methodologies to validated manufacturing controls in bioprocessing. It includes four stages: Discovery Layer (global multi-omics for candidate feature identification), Verification Layer (association of reduced marker sets with biological endpoints), Targeted Assay Layer (implementation of validated PRM/SRM/MRM panels), and CMC/Control Strategy Layer (application of targeted assays for final donor/process monitoring, stability, and impurity control). Created in Biorender. Naryeong Kim. (2026).

This stage-specific framing clarifies why different MS applications sit at different levels of maturity (Figure 2). Targeted gene-editing materials, spent media, and impurity assays are closer to implementation, whereas global omics and single-cell MS methods remain primarily discovery and mechanism-building tools.

While MS offers transformative potential across the CAR-T manufacturing lifecycle, its implementation faces distinct technical and operational challenges at each analytical layer. In the Discovery and Verification Layers, global multi-omics and single-cell MS are heavily constrained by sample-preparation variability, complex computational demands, and batch effects, limiting their utility strictly to offline mechanism building. As candidate markers progress into the Targeted Assay Layer, the challenges shift toward stringent analytical validation; quantitative panels (such as PRM or MRM) require stable isotope-labeled standards, rigorous calibration curves, and careful mitigation of matrix effects to reliably detect low-abundance features. For the CMC and Control Strategy Layer, particularly in-process PAT monitoring, the bottlenecks are largely operational, including the necessity for aseptic sampling integration, prevention of probe fouling, and the need for rapid data turnaround to inform near real-time bioreactor decisions. Finally, for final drug product release, the inherently destructive nature of MS workflows, high cell-input requirements, and sensitivity to pre-analytical variables (such as freeze–thaw history) remain significant hurdles, underscoring why MS is currently positioned to complement rather than replace established rapid release assays.

Crucially, the translational maturity and GMP feasibility of mass spectrometry vary significantly depending on the manufacturing input. For non-cellular starting materials—such as single-guide RNAs, mRNA, and recombinant nucleases, MS workflows have achieved high analytical readiness and are increasingly implemented as formal GMP release. Standardized chromatography separations coupled to high-resolution MS enable routine identity testing, sequence verification, and quantitative evaluation of critical attributes like 5′-capping efficiency and poly(A) tail length distributions. Conversely, for living cellular intermediates and final drug products, MS functions primarily within the discovery, process development, and comparability layers. Implementing multi-omics MS workflows for routine cell-product release remains constrained by operational hurdles, including destructive sampling, high cell-input requirements, sensitivity to pre-analytical sample handling, and multi-day analytical turnaround times that are incompatible with rapid lot disposition. Consequently, MS is positioned to complement established release testing (e.g., flow cytometry, safety assays) by serving as a deep characterization tool during process development, comparability studies, and failed-lot investigations.

## 3. Donor and Leukapheresis: Cellular Starting Material Characterization

Donor and leukapheresis-material characterization is a natural entry point for MS-enabled analytics in allogeneic CAR-T manufacturing because donor-derived starting material can be selected, compared, and characterized before large-scale production. Whereas autologous cell manufacturing relies on patient-derived starting material that is heavily influenced by prior chemotherapy, lymphopenia, and disease burden, allogeneic manufacturing utilizes healthy donor leukapheresis products. Consequently, while autologous MS characterization typically focuses on assessing patient-specific cellular dysfunction or exhaustion baseline, allogeneic donor-stage MS focuses on screening and selecting donor cohorts with optimal baseline immunometabolic fitness to ensure lot-to-lot consistency across large-scale, multi-patient production runs. Importantly, while the biological priorities differ between patient-specific and off-the-shelf paradigms, the stage-specific MS modalities detailed here—including bulk and single-cell proteomics, metabolomics, and surface profiling—serve as universal characterization tools applicable across autologous CAR-T, TCR-T, and donor-derived CAR-NK starting materials. Regulatory donor eligibility testing establishes an essential infectious-disease safety baseline, while CAR-T CMC guidance emphasizes manufacturing control, analytical comparability, and product-specific quality attributes [20,40]. However, these frameworks do not fully define the biological quality of the leukapheresis product. This distinction is important because donor-associated variability can propagate through activation, genetic modification, expansion, harvest, formulation, cryopreservation, and final product performance.

Cellular starting-material composition can influence downstream manufacturing performance. Leukapheresis products differ in T-cell subset composition, accessory-cell content, activation state, proliferative capacity, exhaustion burden, and metabolic readiness, all of which may affect activation response, transduction or editing efficiency, expansion kinetics, final phenotype, potency, and post-thaw recovery [28,41]. MS is therefore best positioned as an orthogonal molecular layer that complements donor eligibility testing, flow cytometry, and functional assays. The most practical implementation relies on a discovery-to-translation pipeline: broad MS fingerprinting during early process development isolates candidate features, which are systematically funneled into robust targeted tracking methods linked to predefined clinical manufacturing endpoints. Crucially, biologically plausible markers should only be adopted operationally if they are supported by analytically robust assays and linked to predefined clinical manufacturing endpoints (Table 1).

### 3.1. Sampling Strategy: Cellular and Systemic Donor Readouts

A practical donor-stage MS workflow can be organized into two complementary layers: a cellular and a systemic donor-state layer. The cellular layer includes PBMCs, enriched T cells, or selected immune-cell subsets from leukapheresis material. Bulk PBMC profiling preserves the mixed immune-cell context of the leukapheresis product, including T cells, B cells, NK cells, monocytes, dendritic cells, and other populations. This broader PBMC context is relevant because PBMC proteomics has provided immune-relevant molecular information from clinical blood samples [42], and leukapheresis composition itself can affect CAR-T manufacturing behavior. For example, depletion of CD14+ monocytes from healthy-donor leukapheresis products has been shown to improve T-cell activation, transduction consistency, CAR expression, cytotoxicity, and final product phenotype compared with unsorted cultures [41].

Conversely, enriched T-cell profiling focuses on the cellular compartment most directly entering CAR-T manufacturing. It is better suited for identifying intrinsic T-cell programs related to activation readiness, differentiation state, mitochondrial function, cytotoxic potential, apoptosis susceptibility, and stress tolerance. Quantitative proteomic profiling of primary human immune-cell populations has shown that immune subsets have distinct proteomic programs in resting and activated states [43], supporting the idea that cellular proteomics can resolve functional immune-cell differences not captured by bulk cell counts alone. These cellular MS readouts should be interpreted alongside conventional measurements, including total cell count, viability, CD4/CD8 ratio, naïve/memory phenotype, activation markers, exhaustion markers, and flow-cytometric identity.

Paired plasma or serum analysis provides a complementary systemic view of the donor-state (Table 1). While cellular MS profiles intrinsic T-cell programs, plasma proteomics and metabolomics capture systemic covariates like inflammatory tone, complement and coagulation activity, nutrient status, and the broader metabolic background [44,45,46,47,48,49].

The usefulness of donor-stage MS depends heavily on matrix-specific sample preparation alongside the chosen acquisition strategy (Table 2). PBMCs and enriched T cells are relatively straightforward for bottom-up proteomics because they can be washed, lysed, reduced, alkylated, enzymatically digested with trypsin or Lys-C/trypsin, and cleaned up prior to LC–MS/MS [50,51]. Low-input or detergent-compatible preparation methods such as SP3 and S-Trap can improve robustness when cell numbers are limited or stronger lysis conditions are required [52,53]. These advanced extraction approaches effectively remove salts, detergents, and other background components that can suppress ionization or reduce quantitative reproducibility.

Plasma and serum present more challenging proteomic matrices because albumin, immunoglobulins, and other high-abundance proteins dominate signals and can mask lower-abundance inflammatory, signaling, or tissue-leakage proteins (Table 2). Depending on the analytical goal, plasma proteomics may therefore require depletion, fractionation, affinity enrichment, nanoparticle-corona enrichment, or targeted workflows [42,44,45]. Broad discovery workflows can increase proteome depth but may introduce added sample-preparation complexity and batch effects, whereas targeted workflows are better suited for quantifying predefined candidates once biologically and analytically justified.

Metabolomics and lipidomics introduce additional analytical vulnerabilities. Because many metabolites are labile, sample handling must minimize enzymatic turnover and degradation through rapid quenching, cold solvent extraction, controlled processing time, and limited freeze–thaw exposure [44,54,55]. Extraction chemistry strongly shapes molecular coverage: polar metabolite workflows enrich amino acids, nucleotides, organic acids, and redox metabolites, whereas lipidomics require organic or biphasic extraction strategies optimized for nonpolar and amphipathic species. No single extraction method captures the full metabolic and lipidomic space without bias. Matrix effects, including ion suppression from salts, proteins, lipids, detergents, and media components, can distort quantification unless controlled through cleanup, dilution, chromatographic separation, stable isotope-labeled standards, pooled QC samples, randomized run order, and batch-correction strategies. These constraints are especially important in donor studies because anticoagulant choice, time to processing, temperature exposure, cell-isolation method, cryopreservation, storage duration, and freeze–thaw history can otherwise be misinterpreted as donor-associated biology rather than sample-handling variation.

### 3.2. Global Cellular Proteomics for Donor-Associated Material Attributes

Using the PBMC or enriched T-cell fractions, global cellular proteomics is the most direct MS approach for identifying donor-associated protein programs in leukapheresis-derived starting material. In a typical bottom-up workflow, cellular proteins are lysed, reduced, alkylated, enzymatically digested into peptides, separated by reversed-phase chromatography, and analyzed by LC–MS/MS. In this context, DDA, DIA, and TMT are acquisition strategies for global proteomics, determining the tradeoff between proteome depth, quantitative reproducibility, and throughput. DDA is useful for early discovery because it can identify abundant peptide features without requiring a fixed target list, but stochastic precursor selection can introduce missing values across donor cohorts. DIA systematically fragments precursor windows and is better suited for larger cohort comparisons where quantitative consistency is important, although it requires more complex computational analysis; tools such as DIA-NN have improved identification depth, interference correction, and reproducibility in high-throughput DIA proteomics [56,57]. Multiplexed isobaric labeling approaches, including TMT, can support controlled donor-to-donor or process-condition comparisons by labeling, pooling, and quantifying samples through reporter ions. However, co-isolation interference can compress fold changes, and MS3 or MultiNotch/SPS-MS3 strategies may be needed to improve quantitative accuracy at the cost of added method complexity [58,59,60].

The rationale for donor-stage cellular proteomics is supported by immune-cell proteomics literature and by CAR-T starting-material studies, although direct CAR-T donor-proteomics evidence remains limited. PBMC proteomics studies demonstrate that donor immune cells can be profiled deeply and reproducibly [42], while quantitative immune-cell atlas studies show that primary immune subsets have distinct proteomic architectures in resting and activated states [43]. These findings support the use of global proteomics to identify donor-associated protein programs related to TCR signaling, cytokine responsiveness, cytotoxic machinery, mitochondrial function, metabolic readiness, apoptosis regulation, senescence, exhaustion, and stress response. In the CAR-T manufacturing context, Noaks et al. demonstrated that leukapheresis composition can influence T-cell activation, transduction consistency, CAR expression, cytotoxicity, and final product phenotype [41]. Together, these studies support the biological plausibility of donor-stage cellular proteomics, but they do not yet establish global proteomics as a validated donor-selection assay for allogeneic CAR-T manufacturing.

For allogeneic CAR-T donor characterization, global cellular proteomics is best positioned as a discovery tool during process development. DDA may support early pilot studies, DIA is better suited for larger donor cohorts where reproducible quantification is critical, and TMT can support multiplexed comparisons when donor samples, process conditions, and batch structure are carefully balanced. However, acquisition time, cell-input requirements, sample-preparation variability, data-analysis complexity, missingness, and batch effects limit immediate operational use. Therefore, global proteomics should be used to nominate candidate donor-associated material attributes for subsequent verification, endpoint association, and targeted assay development.

### 3.3. Plasma and Serum Proteomics as a Systemic Donor-State Layer

Plasma and serum proteomics can complement cellular profiling by capturing systemic donor biology that may contextualize leukapheresis quality. Potentially informative features include complement and coagulation proteins, acute-phase reactants, inflammatory mediators, soluble immune regulators, nutrient-transport proteins, and metabolic-state-associated proteins. These features may not directly define the engineered T-cell product, particularly if plasma carryover is limited during cell processing, but they can identify donor-state covariates that influence, explain, or confound manufacturing performance. This distinction is important for allogeneic CAR-T manufacturing because donors with similar cellular composition may still differ in systemic inflammatory tone, complement activity, coagulation state, or metabolic background.

The major analytical challenge for plasma and serum proteomics is dynamic range. Highly abundant proteins such as albumin, immunoglobulins, transferrin, and other carrier proteins can obscure lower-abundance proteins relevant to inflammation, immune regulation, or stress physiology [44,45,46,47]. This limitation has motivated depletion, fractionation, affinity-enrichment, nanoparticle-corona enrichment, and targeted MS strategies. Blume et al. provide one example of a scalable plasma-proteomics strategy, using multi-nanoparticle protein-corona enrichment to improve depth and throughput in an automated 96-well format [45]. Other plasma-proteomics workflows similarly support cohort-scale profiling and biomarker discovery, but they require careful control of anticoagulant, collection tube, processing time, storage condition, freeze–thaw history, depletion/enrichment strategy, and batch structure [44,46,47,48].

For allogeneic CAR-T manufacturing, plasma or serum proteomics should be framed as a systemic covariate layer rather than as a standalone donor-selection assay. Its greatest value may be in explaining why donors with similar cell counts, subset composition, viability, or naïve/memory phenotype behave differently during activation, genetic modification, expansion, harvest, formulation, or post-thaw recovery. Plasma-derived candidate markers should therefore be tested against defined manufacturing and product-quality endpoints before being incorporated into donor-material attribute panels. In this role, plasma and serum proteomics can strengthen donor and starting-material characterization by adding systemic context to cellular MS, flow-cytometric, and functional readouts.

### 3.4. Metabolomics and Lipidomics of Donor T Cell State

Metabolomics and lipidomics provide functional information that proteomics alone may not capture from donor-derived PBMCs, enriched T cells, or paired plasma/serum. Cellular metabolomics can measure amino acids, nucleotides, organic acids, redox metabolites, acylcarnitines, and other pathway intermediates associated with activation, proliferation, mitochondrial function, and stress adaptation. Lipidomics can profile membrane lipids, sphingolipids, glycerophospholipids, neutral lipids, and lipid mediators that influence membrane organization, signaling, differentiation, and cytotoxic function. These measurements are relevant because T-cell activation and differentiation are tightly linked to metabolic remodeling, and immune-cell function can depend on coordinated changes in nutrient utilization, biosynthesis, redox balance, and membrane composition [61,62,63,64,65].

Several studies support the biological rationale for including metabolomic and lipidomic readouts in donor-stage characterization, although direct evidence for routine metabolomic donor selection in allogeneic CAR-T manufacturing remains limited. Buck, O’Sullivan, and Pearce summarized how T-cell activation, differentiation, and effector function are coupled to metabolic reprogramming [39]. Edwards-Hicks et al. used metabolomics to resolve the metabolic dynamics of in vitro CD8+ T-cell activation, showing that activation is accompanied by complex and time-dependent metabolic changes [62]. More recently, integrated metabolic and proteomic studies have highlighted lipid remodeling as part of T-cell differentiation and function. Kanno et al. identified increased sphingolipid biosynthesis during T-cell differentiation, while Longo et al. showed that glucose-dependent glycosphingolipid biosynthesis supports CD8+ T-cell expansion, cytotoxic function, lipid-raft integrity, and tumor control [66,67,68]. Together, these studies support the broader premise that metabolic and lipidomic state can influence T-cell behavior, even if their use as operational allogeneic CAR-T donor-selection tools remains investigational.

For donor-stage allogeneic CAR-T manufacturing, metabolomics and lipidomics could help identify donor-associated molecular states that are not apparent from cell counts, CD4/CD8 ratio, or naïve/memory phenotype alone. Two donors with similar conventional cellular profiles may differ in amino acid availability, nucleotide metabolism, redox state, acylcarnitine abundance, mitochondrial-associated metabolites, lipid remodeling, sphingolipid abundance, or membrane-associated signaling pathways. These differences could plausibly influence activation response, expansion kinetics, stress tolerance, phenotype stability, cytotoxic potential, or post-thaw recovery. However, metabolomics and lipidomics are especially vulnerable to confounding from diet, fasting state, anticoagulant choice, temperature, time-to-processing, cryopreservation, storage duration, extraction chemistry, and freeze–thaw history [44,48,49,54,55]. Therefore, untargeted metabolomics and lipidomics should be used primarily for discovery, while targeted panels with isotope-labeled standards, calibration curves, pooled QC samples, randomized run order, batch-correction strategies, and prespecified acceptance criteria are more appropriate for translation.

Fluxomics can provide an additional mechanistic layer but is less practical for routine donor screening. Ex vivo stable-isotope labeling can reveal pathway activity, including glucose utilization, glutamine metabolism, nucleotide biosynthesis, redox metabolism, or lipid synthesis [66,69]. By tracing the kinetic movement of labeled carbon and nitrogen skeletons, this methodology maps active pathway transitions rather than simple static baseline pools, providing a valuable mechanistic bridge during media optimization studies.

### 3.5. MS-Enabled and Complementary Single-Cell Phenotyping of Donor Heterogeneity

MS-enabled single-cell phenotyping addresses the limitation that bulk PBMC, or enriched T-cell profiling can average over biologically important subpopulations. In mass cytometry, cells are stained with antibodies conjugated to stable heavy-metal isotopes, nebulized into single-cell droplets, ionized by inductively coupled plasma, and analyzed by time-of-flight MS. This enables simultaneous measurement of dozens of predefined protein markers per cell and can quantify donor-cell subset composition, memory/effector balance, activation state, senescence, exhaustion, and myeloid or accessory-cell populations [67,68]. For allogeneic CAR-T manufacturing, this type of high-dimensional immune phenotyping may help identify rare or disproportionate donor-cell states that are masked by bulk proteomics or conventional cell counts.

The rationale for MS-enabled single-cell phenotyping is supported by foundational mass cytometry and CAR-T-focused studies. Bendall et al. established mass cytometry as a high-dimensional single-cell method for immune and drug-response profiling across the human hematopoietic continuum [67]. Iyer et al. provide practical guidance for CyTOF experiment design, antibody conjugation, staining, acquisition, preprocessing, and analysis [68]. Michelozzi et al. further demonstrated the use of mass cytometry for high-dimensional functional phenotyping of preclinical human CAR-T cells, supporting its relevance to CAR-T analytical development [70]. These studies support CyTOF as a useful donor and product-characterization tool, although it remains antibody-panel-dependent and should be interpreted as targeted single-cell phenotyping rather than unbiased proteomics.

Single-cell functional assays and emerging single-cell LC–MS proteomics can provide additional layers of information, but their maturity differs. Single-cell secretome or microchip-based functional assays can reveal heterogeneity in cytokine secretion, cytolysis, and polyfunctionality among phenotypically similar T cells, making them useful complementary assays even when they are not conventional LC–MS workflows [71,72]. Emerging single-cell LC–MS proteomics can quantify endogenous proteins from individual cells using miniaturized preparation, low-flow chromatography, and high-sensitivity MS, but current limitations include low input, missingness, throughput, technical variability, and data sparsity [73,74]. Imaging mass cytometry and MIBI-TOF extend MS-enabled phenotyping into spatial tissue context, although their relevance is stronger for tumor microenvironment studies or post-infusion translational analyses than for routine leukapheresis screening [75,76].

For donor-stage allogeneic CAR-T manufacturing, these approaches should be positioned as complementary tools rather than replacements for conventional flow cytometry or bulk LC–MS proteomics. CyTOF is the most mature MS-enabled single-cell approach for high-dimensional immune phenotyping, while single-cell LC–MS proteomics remains an emerging discovery technology. Together, these methods may help determine whether donor leukapheresis products contain subpopulations that support or impair activation, genetic modification, expansion, cytotoxic differentiation, stress tolerance, or post-thaw recovery. However, any single-cell-derived candidate markers should still be linked to manufacturing endpoints before being incorporated into donor-material attribute panels.

### 3.6. Targeted Proteomics for Donor Comparability Panels

Targeted proteomics is the translational step that converts discovery markers into defined, quantitative assays. Reproducible donor-associated features identified through global cellular proteomics, plasma profiling, metabolomics-linked pathway analysis, or biological prior knowledge can be translated into targeted MS assays such as MRM/SRM, PRM, or triggered PRM. In MRM or SRM workflows, proteotypic peptides are selected for each candidate protein, stable isotope-labeled internal standards are added, and scheduled precursor-to-fragment ion transitions are monitored on a triple-quadrupole instrument. In PRM, target precursor ions are isolated on a high-resolution instrument and full fragment-ion spectra are collected, improving selectivity and simplifying method development for moderate-sized panels [77,78,79]. Internal standard-triggered PRM can further improve duty-cycle efficiency by triggering acquisition based on the presence of spiked standards, providing a route toward larger targeted panels [80].

Both MRM/SRM and PRM typically incorporate stable isotope-labeled internal standards, calibration strategies, and predefined QC criteria, allowing discovery candidates to be converted into fixed quantitative panels for donor comparability or starting-material characterization [19,77,78,79,80]. The feasibility of targeted MS for complex biological matrices is supported by foundational plasma and proteomics studies. Kuzyk et al. demonstrated multiplexed absolute quantitation of 45 proteins in human plasma using MRM [77], while Peterson et al. and Rauniyar describe PRM as a high-resolution targeted strategy with strong selectivity and specificity [78,79]. Gallien et al. extended this concept through internal standard-triggered PRM, highlighting a path toward larger targeted panels [80]. Boja et al. provide analytical-validation considerations for multiplex MS biomarker platforms, including specificity, precision, sensitivity, linearity, reproducibility, and robustness [19]. More formal validation frameworks, such as CLSI guidance for quantitative protein and peptide measurement by MS, can further strengthen the path from discovery marker to operational assay.

For allogeneic CAR-T donor characterization, targeted proteomics is the most plausible route from discovery profiling to implementation. Candidate markers identified by global proteomics, plasma profiling, metabolomics-linked pathway analysis, or biological prior knowledge could be converted into panels that monitor memory-associated proteins, activation burden, cytotoxic potential, stress response, complement or inflammatory covariates, or other donor-associated material attributes. However, targeted panels should not be built from biological plausibility alone. A donor panel must be anchored to manufacturing and product-quality endpoints such as activation response, transduction or editing efficiency, expansion kinetics, viability, phenotype, potency, or post-thaw recovery. Otherwise, the result may be an analytically robust assay with limited operational value.

A practical translation pathway would include four stages. First, broad discovery profiling identifies candidate donor-associated molecular features. Second, those candidates are tested against prespecified manufacturing and product-quality endpoints. Third, reproducible candidates are converted into targeted MRM, PRM, triggered PRM, or LC–MS/MS panels with stable isotope-labeled standards, calibration strategies, QC criteria, and acceptance thresholds. Fourth, the resulting assays are evaluated prospectively across donor lots, manufacturing runs, and process changes to determine whether they support donor comparability, starting-material risk assessment, or process-development decision-making. This staged model keeps MS aligned with the central CMC question: whether a molecular measurement improves understanding, comparability, or control of starting-material variability in a way that is actionable for allogeneic CAR-T production.

## 4. Mass Spectrometry for the Characterization of Gene Editing Starting Materials

The transition from autologous, patient-specific CAR-T therapies to allogeneic, “off-the-shelf” platforms requires precise, multiplexed genetic modifications of healthy donor T cells to prevent alloreactivity and enhance therapeutic persistence [81]. Critical edits include disruption of the TRAC or TRBC genes to eliminate T-cell receptor expression and prevent GvHD, knockout of B2M to evade host immune rejection, and knockout of CD52 to confer resistance to alemtuzumab-based lymphodepletion. Additional functional edits, such as PDCD1 disruption to reduce T-cell exhaustion or CD7 knockout to prevent self-killing in CD7+ malignancies, are implemented depending on the therapeutic design [30]. These multiplexed edits are achieved using highly defined genome editing platforms whose components directly dictate the CQAs of the final CAR-T product.

While early allogeneic platforms relied on protein-based nucleases such as TALENs or ARCUS meganucleases, CRISPR/Cas9 has become the dominant architecture due to its structural simplicity and flexibility with single-guide RNAs [82]. The resulting ribonucleoprotein (RNP) complexes provide transient, kinetically controlled editing, offering rapid on-target activity while minimizing prolonged cellular exposure. Delivery of the nuclease via mRNA provides an additional transient expression modality, avoiding the risks of random genomic integration associated with DNA vectors [83]. However, ensuring the safety and efficacy of these genome editing platforms shifts a massive regulatory and analytical burden onto the starting materials themselves, requiring multi-attribute characterization workflows capable of detecting subtle stereochemical, sequence, and structural variants.

Although lipid nanoparticles (LNPs), viral vectors, and other gene-delivery systems represent critical enabling technologies for intracellular transport, a comprehensive discussion of their analytical characterization falls beyond the scope of this review. Similarly, the detailed evaluation of lipid excipients and nanoparticle physicochemical attributes has been extensively detailed [84,85,86]. To address the fundamental molecular building blocks of modern cell engineering, this section critically evaluates mass spectrometry applications for the direct, high-resolution characterization of gene-editing starting materials themselves: sgRNAs, mRNA, nucleases, RNP complexes, and DNA transgene delivery templates. While non-edited autologous CAR-T products may only require analytical characterization of viral delivery vectors, allogeneic platforms rely heavily on these multiplexed gene-editing inputs. Nevertheless, the intact mass, sequence mapping, and impurity LC-MS workflows described here for gene-editing reagents represent standard analytical blueprints applicable to any gene-edited cell or gene therapy platform.

### 4.1. Single-Guide RNAs

High-resolution mass spectrometry provides definitive confirmation of molecular mass, sequence-related impurities, chemical modifications, and degradation products for gRNAs, which typically span approximately 100 nucleotides. For these full-length frameworks, ion-pair reversed-phase liquid chromatography (IP-RPLC) coupled to electrospray ionization mass spectrometry (ESI-MS) represents the standard analytical paradigm (Table 3). Utilizing volatile alkylamine/hexafluoroisopropanol ion-pairing agents paired with C18 or Charged Surface Hybrid m stationary phases, this configuration resolves closely related synthesis impurities, including truncated failure sequences (e.g., N-1, N-2), desulfurization products, and chemical adducts. A vital utility of this framework is the evaluation of N + 1 insertions, which frequently share an almost identical mass-to-charge ratio (*m*/*z*) envelope with the target sgRNA. High-resolution chromatographic selectivity isolates these nearly isobaric species prior to MS analysis based on subtle differences in hydrophobicity or base composition, preventing the impurity from co-eluting with and masking the target drug profile [87].

To probe internal structural features without prior enzymatic cleavage, top-down MS utilizes gas-phase fragmentation techniques such as collision-induced dissociation (CID) or higher-energy collisional dissociation (HCD) [88]. Fragmenting the intact sgRNA within the mass spectrometer generates a comprehensive map of product ions (*a*, *w*, *c*, *d* series), enabling localized mapping of specific chemical modifications (e.g., 2′-O-methyl or phosphorothioate linkages) and pinpointing single-base spacer substitutions directly within the full-length framework. However, top-down MS spectra of 100-mers suffer from severe peak congestion and overlapping charge states, which historically capped sequence coverage (Table 3).

Coupling top-down workflows with cyclic ion mobility spectrometry (cIMS) addresses this constraint by adding a rapid, millisecond-scale gas-phase separation based on molecular size, shape, and charge (electrophoretic drift time) [89]. This gas-phase fractionation resolves highly congested fragment ion envelopes prior to mass analysis. By physically dispersing overlapping isotopic distributions, cIMS dramatically reduces spectral noise and uncovers previously obscured, high-confidence fragment assignments across heavily modified internal guide regions, expanding sequence coverage from a modest 61% to over 95% for intact 100-mer formats [89].

A major analytical hurdle in heavily modified sgRNAs is characterizing phosphorothioate (PS) linkages. Replacing a non-bridging oxygen atom with a sulfur atom introduces a chiral center at the phosphorus atom, yielding two distinct stereoisomeric configurations: the *R*_p_ and *S*_p_ diastereomers. Because standard chemical synthesis is non-stereospecific, an sgRNA containing multiple PS modifications exists as a complex mixture of diastereomers that possess identical elemental compositions and identical *m*/*z* values, causing them to co-elute during standard LC workflows. The integration of high-resolution ion mobility mass spectrometry (IM-MS) utilizing structures for lossless ion manipulation (SLIM) or cIMS overcomes this limitation by determining an analyte’s experimental collision cross section (CCS) [90,91,92]. Because the R_p_ and S_p_ configurations introduce subtle stereochemical asymmetry that alters the spatial orientation and gas-phase folding of the oligonucleotide backbone, they exhibit distinct aerodynamic drag properties in a mobility drift cell. High-resolution IM-MS capitalizes on these micro-conformational variations to resolve stereoisomeric impurities—both at the intact sgRNA level and within highly complex short digest fragments [90,91,92]. This gas-phase separation yields unique CCS fingerprint profiles, offering an indispensable regulatory framework for tracking batch-to-batch stereochemical uniformity, which is critical since R_p_ and S_p_ configurations can display divergent resistance to endogenous nucleases.

While intact oligonucleotide MS analysis is traditionally performed under denaturing, negative ion mode conditions to track the primary sequence, native mass spectrometry (nMS) frequently utilized in positive ion mode—offers an alternative for probing higher-order structures (HOS), non-covalent aggregation, and complex structural impurities. By utilizing volatile, near-neutral pH buffers (such as ammonium acetate) that preserve native secondary and tertiary folding, nMS detects self-associated structures, hairpins, and sgRNA-protein complexes (e.g., sgRNA bound to Cas9).

**Figure 3:**
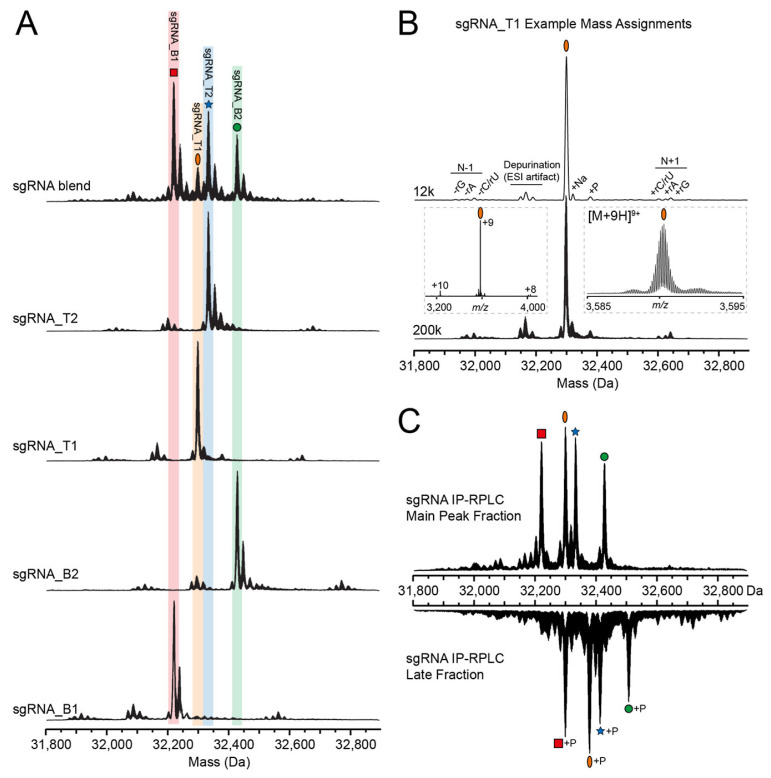
Native positive-ion mode static spray ESI-MS characterization of intact sgRNA. (**A**) Deconvoluted mass spectra of the multiplexed sgRNA pool alongside each individual sgRNA construct. (**B**) Comparative resolution of the sgRNA_T1 analyte between 12k (average mass) and 200k (isotopically resolved) mass resolution; insets illustrate the native charge-state distribution envelope and an expanded view of the baseline-resolved +9 charge state. (**C**) Comparative analysis of the primary IP-RPLC peak and late-eluting fractions, revealing an identical spectral profile shifted by +80 Da, confirming a terminal or internal phosphorylation variant (adapted from [93]).

Recent work integrating high-resolution size-exclusion chromatography (SEC) and IP-RPLC fractionation with native MS has established clear relationships between macro-structural sgRNA attributes and downstream functional impacts [93] (Figure 3). This workflow deeply profiles physical variants, such as non-covalent multimeric aggregates, early eluting truncation impurities, and phosphorylated variants. Crucially, native MS-verified sgRNA aggregates exhibit a 10% reduction in knockout efficiency alongside increased cumulative off-target editing frequencies, while target-specific deletion impurities yield sharp drops in gene-editing performance in cellular systems [93]. However, nMS faces a distinct limitation: while they yield high-quality MS spectra with high sensitivity and low sample requirements (e.g., 10-µg), the offline buffer-exchange requirement for salt removal restricts high-throughput analysis. To address this bottleneck, the path forward relies on integrating advanced separation techniques directly with native MS. The online coupling of native-compatible workflows—such as volatile SEC—is anticipated to eliminate manual descaling steps and further expand the high-throughput detection of sgRNA modifications and structural variants.

For unequivocal sequence confirmation, precise detection of intended modifications, and screening of trace impurities, oligonucleotide mapping (bottom-up analysis) is employed following sequence-specific enzymatic digestion using specialized endonucleases [94]. Beyond mapping, this methodology is vital for Sequence Variant Analysis (SVA) to track low-abundance impurities, such as unintended single-nucleotide polymorphisms or point mutations introduced during production (e.g., a guanosine-to-adenosine transition). While intact mass analysis identifies major mass shifts, it often fails to pinpoint these low-abundance sequence variants or positional isomers where a modification is placed on the wrong nucleotide.

Following enzymatic digestion, the resulting oligonucleotide fragments provide a high-resolution representation of the sgRNA sequence and can be further characterized using chromatographic separation coupled with high-resolution MS. This integrated workflow enables comprehensive assessment of sequence integrity, modification placement, and impurity profiles, thereby complementing intact mass analysis and enhancing confidence in sgRNA quality attributes.

The resulting short digested fragments are subsequently separated using either IP-RPLC or hydrophilic interaction liquid chromatography (HILIC) prior to mass analysis [94,95]. While IP-RPLC is highly effective, HILIC offers distinct advantages for MS compatibility in bottom-up workflows [96]. Because HILIC separates hydrophilic analytes using a high organic solvent starting mobile phase (typically acetonitrile) without the need for non-volatile or MS-suppressing ion-pairing agents, it significantly enhances desolvation efficiency in the ESI source [95,96]. This results in drastically reduced ion suppression and superior MS sensitivity for small, highly polar digested fragments, while eliminating the arduous column-flushing protocols associated with ion-pairing systems [96]. To translate these complex data streams into actionable results, automated deconvolution algorithms allow precise assignment of complex, highly charged state envelopes and the detection of low-abundance impurities. Together, these orthogonal MS strategies provide a robust platform for ensuring the sequence integrity, modification fidelity, and chemical consistency of sgRNA therapeutics.

### 4.2. Messenger RNAs

Extending LC-MS workflows to mRNA requires a fundamental shift in the analytical paradigm [97]. In vitro transcribed mRNAs present a more complex analytical challenge than sgRNAs due to their immense macromolecular size, typically ranging from 1000 to several thousand nucleotides. A premier example in CAR-T manufacturing workflows is the Cas9 mRNA construct; with a massive size of approximately 4500 nucleotides, its characterization is particularly complicated. Because intact mass measurements of such full-length mRNAs far exceed the practical resolution limits of conventional ESI-MS owing to extreme charge-state dispersion and signal attenuation, characterization frameworks pivot toward targeted bottom-up oligonucleotide mapping and site-specific enzymatic clipping strategies [98,99].

By utilizing sequence-specific endonucleases—most notably RNase T1, which selectively cleaves the phosphodiester backbone at the 3′ end of guanosine residues—the mRNA is digested into a reproducible pool of short fragments [100]. When coupled to LC-MS via the IP-RPLC or HILIC configurations established in the previous section, this mapping paradigm achieves high sequence coverage (e.g., 70–90%). This high-density sequence verification confirms primary sequence fidelity, maps the precise positional incorporation of critical chemical modifications such as N1-methylpseudouridine, and distinguishes the target product from low-abundance transcriptional variants or template-derived DNA contamination [98,101].

**Figure 4:**
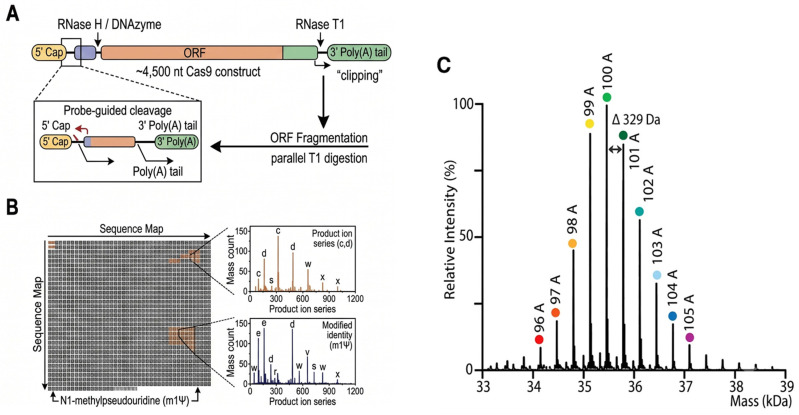
**Multi-attribute liquid chromatography-mass spectrometry (LC-MS) framework for the comprehensive characterization of long therapeutic IVT mRNA constructs.** (**A**) Schematic of the dual-track enzymatic processing strategy: site-specific, probe-guided DNAzyme or RNase H cleavage isolates the functional 5′ Cap and 3′ Poly(A) tail domains, while parallel, complete RNase T1 digestion fragments the large internal ORF. (**B**) High-density bottom-up oligonucleotide mapping grid of the internal ORF sequence. Tandem mass spectrometry (MS/MS) product-ion fragment footprints provide high sequence coverage, verifying primary sequence identity and localizing the exact site-specific percentage incorporation of critical chemical modifications. (**C**) Mass profile for the analysis of the 120-Poly(A) tail. The increment of an adenosine residue (+329 Da) is represented by different colored dots. Figure 4C is adapted from [104].

Beyond overall sequence mapping, HRMS can be employed to monitor the distinct CQAs at the mRNA termini, which dictate in vivo stability and translational efficiency (Table 3). At the 5′ terminus, the presence of a proper cap structure (typically Cap 1) prevents exonuclease degradation and promotes ribosome loading [102]. Because the capped 5′ fragment represents a minor, highly specific component of the total digest, researchers utilize targeted cleavage strategies—often employing DNAzymes or RNase H guided by complementary oligonucleotides—to selectively liberate the 5′ terminal fragment (typically a 10- to 20-mer) while leaving the downstream sequence intact [97] (Figure 4). When analyzed via ESI-MS, this targeted approach allows absolute quantification of capping efficiency and differentiates functional Cap 1 from un-capped (5′-triphosphate or 5′-hydroxyl) or partially capped (Cap 0) species.

Concurrently, mass spectrometry characterizes the 3′ polyadenylation [poly(A)] tail, a homopolymer sequence crucial for modulating mRNA half-life. Standard biophysical techniques like capillary electrophoresis yield only broad, apparent sizing distributions of the poly(A) region due to inherent resolution constraints. Mass spectrometry addresses this bottleneck by isolating the poly(A) region through sequence-specific cleavage and analyzing the released tail by high-resolution LC-MS, enabling accurate measurement of poly(A) tail length and heterogeneity (Figure 4) [99]. This approach yields direct, nucleotide-resolution mass profiles of the poly(A) tail length distribution. Crucially, high-resolution MS mapping detects trace sequence variants within the tail itself, such as the accidental incorporation of non-adenine nucleotides during enzymatic IVT production, providing a rigorous window into the transcriptional consistency of the poly(A) polymerase used during synthesis.

Gas-phase separation techniques like IM-MS further elevate mRNA mapping, particularly for isolating isobaric digest products and structural isomers (Table 3). During complex multi-enzyme digestion of large mRNAs, many short oligonucleotide fragments possess identical elemental compositions but divergent sequences or distinct internal modification patterns (positional isomers). Incorporating cIMS or SLIM allows these co-eluting, isobaric fragments to be rapidly separated based on their unique *CCS* values before entering the mass analyzer, mirroring the resolution of stereochemical anomalies seen in smaller RNA therapeutics [103].

Crucially, native mass spectrometry workflows map macro-structural, non-covalent, and self-complementary variants that escape standard denaturing LC-MS protocols. Recent benchmark studies profiling therapeutic IVT mRNA batches demonstrated that severe immunogenic impurities are predominantly driven by single-stranded truncation variants with incomplete poly(A) architectures, alongside highly stable dsRNA byproducts generated via self-priming or antisense transcription. By implementing nMS, researchers have successfully resolved these highly elusive dsRNA anomalies, identifying the problematic 3′-loopback dsRNA form at single-nucleotide resolution [104]. This precise structural localization under native spray states links specific structural configurations to cell-based immune responses, providing process developers with the mechanistic target required to mitigate immunogenicity during the T7 RNA polymerase transcription phase [105]. Combined with sophisticated algorithmic deconvolution software capable of handling heavily obscured charge envelopes [106], modern native MS and LC-IM-MS/MS platforms provide a remarkably robust framework for checking the overall structural architecture, sequence integrity, and batch-to-batch uniformity of long-chain therapeutic mRNA constructs [107].

### 4.3. Genome Editing Nucleases

Genome editing nucleases—including Cas9, Cas12a, TALENs, and engineered base or prime editors—are critical protein starting materials whose structural integrity directly influences editing efficiency, specificity, and cellular safety. High-resolution mass spectrometry evaluates nuclease identity, purity, post-translational modifications (PTMs), conformational integrity, and degradation pathways. To minimize off-target cleavage, wild-type enzymes are increasingly replaced by engineered high-fidelity variants, such as SpyFi Cas9. Confirming the precise primary sequence and PTM profile of these variants is vital for regulatory approval, often requiring the harmonization of multi-attribute mass spectrometry pipelines with orthogonal capillary electrophoresis (CE) frameworks like CE-SDS and imaged capillary isoelectric focusing (icIEF) to track sizing variants and isoelectric points [108].

Intact protein mass analysis using denaturing ESI-MS rapidly confirms molecular weight and detects heterogeneous proteoforms, including truncations, oxidation, deamidation, and non-specific adducts generated during recombinant expression [109]. Because Cas9 proteins are large, multidomain enzymes (~160 kDa), coupling intact mass measurements with reversed-phase liquid chromatography or SEC-MS reduces spectral complexity and improves deconvolution accuracy. These workflows monitor aggregation, fragmentation, and storage-induced degradation during process development and stability studies to mitigate immunogenic risks or compromised editing activity.

For comprehensive sequence verification and PTM localization, bottom-up peptide mapping by LC-MS/MS remains the primary analytical strategy. While traditional trypsin digests are standard, targeted enzymatic strategies are required to differentiate highly homologous engineered variants from their wild-type counterparts. For instance, peptide mapping based on LC-UV-MS/MS using endoproteinase Lys-C under non-reducing conditions provides a powerful framework for absolute amino acid sequence confirmation, allowing the definitive differentiation of SpyFi Cas9 from wild-type SpCas9 based on unique peptide fingerprinting [108].

Following enzymatic digestion (e.g., via trypsin, Lys-C, or Glu-C), tandem mass spectrometry maps amino acid sequence coverage and characterizes site-specific oxidation, deamidation, glycation, disulfide scrambling, and free cysteines [109]. Oxidative damage within catalytic domains or nucleic acid-binding interfaces can alter nuclease folding, reduce cleavage efficiency, and influence off-target editing behavior. Multi-attribute monitoring workflows integrating automated peptide mapping and quantitative LC-MS analysis allow simultaneous monitoring of multiple CQAs within genome editing nucleases during manufacturing lot-to-lot assessments.

Hydrogen-deuterium exchange mass spectrometry (HDX-MS) expands this structural toolbox by interrogating protein conformational dynamics and nucleic acid-induced structural rearrangements. In HDX-MS, solvent-accessible backbone amid hydrogens exchange deuterium, mapping flexible and protected regions throughout the nuclease structure. Comparative HDX-MS studies demonstrate that sgRNA binding induces large-scale conformational stabilization within the recognition and nuclease lobes of Cas9, while engineered high-fidelity variants exhibit altered dynamic behavior near DNA-binding interfaces associated with improved specificity [110,111]. These measurements provide mechanistic insight linking protein structural dynamics directly to editing fidelity and functional performance.

### 4.4. Ribonucleoprotein Complexes

The functional genome editing entity in many transient workflows is the assembled ribonucleoprotein complex formed between the nuclease and sgRNA. These RNP assemblies constitute dynamic macromolecular structures whose stoichiometry, conformational integrity, and assembly efficiency directly impact editing kinetics, intracellular persistence, and off-target activity [112]. Consequently, analytical characterization of RNP complexes is essential within CMC strategies for ex vivo edited cell therapies.

Native mass spectrometry is uniquely suited for direct interrogation of intact RNP assemblies because it resolves intact Cas9-sgRNA complexes according to their true assembly state (Table 3). This allows clear differentiation among apo-nuclease, partially loaded intermediates, and fully assembled RNP species [113]. When coupled to high-resolution Orbitrap or time-of-flight analyzers, nMS determines complex stoichiometry while simultaneously monitoring low-abundance aggregates, dissociation products, or misassembled species that escape conventional biophysical assays.

The incorporation of IM-MS further enhances RNP characterization by providing gas-phase conformational separation prior to mass analysis. CCS measurements generated through cIMS or SLIM workflows discriminate conformational subpopulations and partially unfolded intermediates that possess identical molecular masses but distinct three-dimensional architectures [114]. This additional structural dimension evaluates engineered Cas variants or chemically modified sgRNAs whose altered conformational dynamics influence target engagement and editing specificity.

Cross-linking mass spectrometry (XL-MS) serves as a complementary structural biology tool for mapping protein-RNA interaction interfaces within these RNP assemblies. By covalently stabilizing proximal amino acid and nucleotide interactions prior to proteolytic digestion and LC-MS/MS analysis, XL-MS identifies direct contact regions between Cas nucleases and guide RNAs [115]. These measurements provide mechanistic insight into sgRNA recognition, conformational activation, and engineered domain rearrangements in next-generation platforms such as base editors and prime editors.

### 4.5. Double- and Single-Stranded DNA Transgene Templates

Compared with smaller oligonucleotides and transcripts, intact double-stranded DNA (dsDNA) plasmids and single-stranded DNA (ssDNA) templates (~1–20 kb) used to deliver large CAR transgene sequences present distinct analytical challenges due to their megadalton-scale macromolecular size and topological heterogeneity. Low-level sequence mutations, truncation variants, or promoter rearrangements can compromise downstream transfection efficiency and lead to variable CAR expression kinetics in therapeutic cells.

Because full-length dsDNA plasmids far exceed the practical resolution limits of conventional electrospray platforms, sequence validation relies on targeted bottom-up liquid chromatography-mass spectrometry following sequence-specific restriction enzyme digestion or controlled nuclease cleavage [99]. Operating as MAM, this bottom-up configuration simultaneously confirms primary sequence fidelity across the CAR open reading frame, quantifies single-nucleotide polymorphisms down to 0.1%, maps exonuclease-shielding phosphorothioate modifications, and screens for co-purified host-cell contaminants in a single run [116]. Beyond primary sequence auditing, monitoring the macrostructural 3D topology of a CAR plasmid lot is a vital regulatory requirement, as cellular transfection efficiency and transcriptional productivity heavily depend on maintaining a high percentage (>80–90%) of the tightly coiled supercoiled form rather than the relaxed circular or open-linear degradation topologies. To bypass the resolution limits of bulk, low-resolution biophysical assays (such as agarose gel electrophoresis), Charge Detection Mass Spectrometry (CD-MS) has emerged as a powerful single-particle paradigm (Table 3). By simultaneously measuring the mass-to-charge ratio (*m*/*z*) and the absolute charge of individual megadalton electrospray ions, CD-MS determines true molecular mass independently of bulk calibration standards, providing an analytical resolution unachievable by standard ESI-MS due to extreme charge-state overlapping.

**Figure 5:**
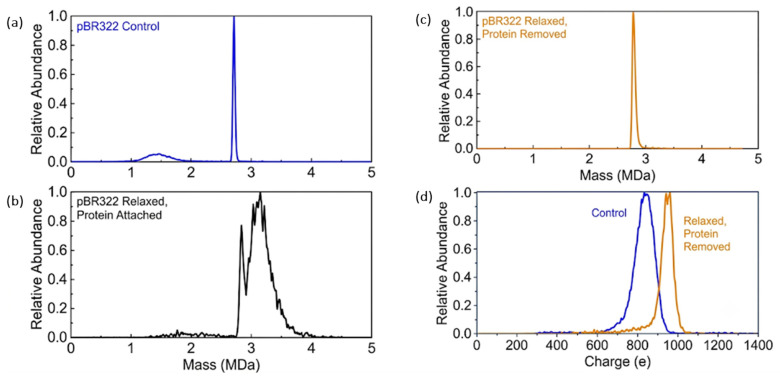
**Single-particle macromolecular auditing of intact dsDNA plasmid vectors using CD-MS.** Panels (**a**–**c**) plot relative abundance against the true calculated molecular mass in megadaltons (MDa) for a pBR322 plasmid system. Panel (**a**) reveals the pristine control standard containing the primary supercoiled population (~2.74 MDa), panel (**b**) tracks structural shifts during topoisomerase-mediated relaxation with protein components transiently bound, and panel (**c**) confirms the baseline mass retention after complete enzymatic relaxation and protein removal. Crucially, panel (**d**) demonstrates the core regulatory value of the technique: while the supercoiled control (blue) and the relaxed, open-circular form (orange) possess an identical true molecular mass, they are separated into distinct, non-overlapping profiles based on absolute charge. The relaxed plasmid exhibits a significant upward charge distribution shift driven by its extended, open gas-phase folding orientation [117].

Recent benchmarks demonstrate that the principal plasmid structural types possess unique, distinguishable charging profiles driven by their spatial orientation and gas-phase folding trajectories [117]. Under native positive-mode electrospray ionization, supercoiled CAR plasmids exist in open configurations that deposit a high charge footprint, which condenses into a low-charge population upon polycation-induced compaction (Figure 5). Crucially, enzymatic alterations to topology yield drastic, predictable charge distribution shifts: relaxing a supercoiled plasmid into a circular state transitions the analyte to higher charge distributions, while complete double-stranded linearization generates an even more pronounced charge increase [117]. By correlating aerodynamic charge deposition with macrostructural folding, CD-MS provides an indispensable framework for direct topological auditing, ensuring batch-to-batch uniformity of critical macromolecular transgene delivery templates.

## 5. MS-Based In-Process Monitoring and PAT During CAR T-Cell Expansion

CAR T-cell products are dynamic living therapies whose quality is shaped by activation, gene modification, expansion, formulation, and cryopreservation. During ex vivo expansion, nutrient availability, waste accumulation, cytokine exposure, oxygenation, and culture duration can influence cell yield, viability, differentiation state, exhaustion, and functional potency. T-cell activation and differentiation are tightly coupled to metabolic remodeling, including changes in glycolysis, mitochondrial metabolism, amino acid utilization, nucleotide biosynthesis, redox balance, and lipid metabolism [118]. These dependencies make molecular and metabolic monitoring particularly relevant for allogeneic CAR T-cell manufacturing, where scalable production, lot-to-lot consistency, and robust process control are central goals.

Process analytical technology provides a framework for measuring process-relevant variables during manufacturing rather than relying exclusively on endpoint characterization. In CAR T-cell expansion, the most practical near-term application of MS-enabled PAT is targeted analysis of spent media, where defined nutrients and metabolites can be monitored over time to support feed strategy, media exchange, harvest timing, and detection of process drift (Table 4). By contrast, untargeted metabolomics, isotope-tracing fluxomics, secretome proteomics, surface proteomics, and lipidomics are better positioned for process development, mechanism discovery, comparability assessment, and candidate CQA identification. Commercial at-line and online platforms such as REBEL/REBEL XT and MAVEN illustrate how analytical monitoring can be brought closer to the bioreactor, although these systems should be described as targeted monitoring platforms rather than comprehensive real-time omics technologies [18,119,120,121].

### 5.1. Targeted Metabolite Monitoring of Spent Media

Targeted metabolite monitoring is one of the most actionable analytical approaches for in-process CAR T-cell culture monitoring (Table 4). The REBEL cell culture media analyzer was introduced as a miniature CE–MS-based at-line platform for spent-media analysis, enabling near-bioreactor measurement of defined media components rather than relying exclusively on centralized analytical laboratories [18]. Earlier REBEL materials described measurement of more than 30 media components, including amino acids, dipeptides, water-soluble vitamins, and amines [120]. More recent REBEL XT materials describe at-line analysis of 19 amino acids, 2 dipeptides, and choline, with rapid reporting from small-volume samples [121].

In CAR T-cell expansion, these measurements could support feed adjustment, media exchange, harvest timing, and detection of process drift by tracking nutrient depletion or metabolite accumulation over time. However, REBEL-style systems should be described as targeted media analyzers, not comprehensive metabolomics platforms. Glucose consumption and lactate accumulation are also important culture-state indicators, but they may require complementary tools. For example, MAVEN is positioned as an online glucose/lactate analyzer using diffusion sampling technology, with optional automated substrate-feed control [119]. Thus, a practical monitoring strategy may combine targeted MS-based nutrient profiling with complementary online glucose/lactate sensors.

### 5.2. Untargeted Metabolomics and Fluxomics for Process Development

Untargeted LC–MS and GC–MS metabolomics can provide broader intracellular and extracellular metabolic profiles during process development (Table 4). Edwards-Hicks et al. profiled metabolic dynamics during in vitro CD8+ T-cell activation and showed that activated CD8+ T cells undergo complex metabolic remodeling that can be resolved using complementary metabolomics approaches [62]. Similarly, Culberson et al. used at-line intracellular analysis to identify spectral and metabolic features associated with T-cell activation state, supporting the use of metabolomics for early detection of T-cell state changes during process development [122].

Stable-isotope tracing provides a mechanistic layer by measuring nutrient routing rather than only metabolite abundance. Longo et al. showed that glucose-dependent glycosphingolipid biosynthesis supports CD8+ T-cell expansion, cytotoxic function, lipid raft aggregation after TCR stimulation, and tumor control [65]. In CAR T-cell process development, analogous tracing maps show how carbon shifts into core biosynthetic pathways based on specific cytokine feeding regimes, establishing a definitive biochemical framework for rational media screening and bioreactor loop engineering.

### 5.3. Secretome Proteomics

Culture supernatant proteomics can complement ELISA or bead-based cytokine assays by enabling multiplexed, antibody-independent measurement of selected secreted proteins. For example, LC–MS/MS MRM assays have been developed to quantify proteins secreted by primary human cells, including assays with calibration across wide dynamic ranges and low abundance detection [123]. Other antibody-free MRM methods have quantified panels of human cytokines in cellular experiments, demonstrating that targeted MS can be applied to secreted immune mediators when enrichment, internal standards, and assay validation are carefully designed [124,125].

For CAR T-cell process development, these approaches could support predefined panels of cytokines, chemokines, stress-associated proteins, or process-related proteins in culture supernatants. However, many cytokines are low abundance, and LC–MS-based secretome profiling can be limited by sensitivity, matrix effects, dynamic range, and sample-preparation requirements. Therefore, MS-based secretome profiling should be framed as complementary to established immunoassays, not as a near-term replacement for routine cytokine release testing.

### 5.4. Cell-Surface and Cellular Proteomics

Cell-surface proteomics provides a concrete example of how MS can capture T-cell phenotypes beyond predefined flow-cytometry panels. Byrnes et al. used quantitative cell-surface capture glycoproteomics to profile primary human T cells and found that activation, regulatory T-cell coculture, and hypoxia induce bidirectional remodeling of the CD8+ T-cell surfaceome [126]. Hypoxia produced particularly strong changes in the activated CD8+ surface proteome and altered nutrient transporter expression, linking surface phenotype to metabolic and immunosuppressive stress [126].

For CAR T-cell manufacturing, analogous workflows could compare activation reagents, cytokine strategies, media formulations, oxygenation conditions, or culture durations by measuring surface proteins associated with memory state, activation, exhaustion, homing, adhesion, nutrient uptake, and differentiation. Broader cellular proteomics could also capture signaling, stress-response, metabolic, and cytotoxicity-associated programs. These methods are best positioned for process characterization, comparability studies, and CQA discovery because they require cell harvest, enrichment or digestion, LC–MS/MS acquisition, and computational analysis.

### 5.5. Lipidomics and Membrane Composition

Lipidomics has been used directly to study T-cell activation and differentiation. Kanno et al. integrated metabolic and proteomic data to examine T-cell differentiation and performed cellular lipidomics after TCR stimulation. Their analysis identified 567 lipid species across cholesterol, free fatty acids, glycerolipids, lysophospholipids, phospholipids, and sphingolipids, and showed that TCR stimulation substantially altered lipid composition during helper T-cell differentiation [63]. This provides a concrete example of how lipidomics can resolve activation-associated membrane and metabolic remodeling in T cells.

Additional studies connect lipid metabolism to T-cell function under stress and antitumor activity. Hunt et al. used metabolic, lipidomic, and imaging approaches to show that acetyl-CoA carboxylase (ACC) activity promotes lipid storage in CD8+ T cells in the tumor microenvironment and that limiting ACC activity supports energy maintenance, persistence, and polyfunctionality under stress [64]. Longo et al. further showed that glucose-dependent glycosphingolipid biosynthesis supports CD8+ T-cell expansion, cytotoxic function, lipid raft aggregation, and tumor control [65]. By tracking these structural wall transitions, lipidomic profiling maps how physical membrane structures adapt to scale-up demands, revealing critical changes in membrane fluidity and signaling raft clustering that are invisible to standard fluid protein assays.

### 5.6. Automation, Data Integration, and Process Control

For MS-based monitoring to support PAT, assay speed, sampling automation, and data interpretation must align with manufacturing decision timelines. REBEL and REBEL XT illustrate at-line CE–MS media analysis for defined nutrients, while MAVEN illustrates complementary online glucose/lactate monitoring and automated feeding control [18,119,120,121]. These examples support a practical near-term model in which targeted MS provides richer media-component profiles, while complementary online sensors provide high-frequency monitoring of key metabolites such as glucose and lactate.

The long-term value of MS-enabled PAT will depend on linking molecular readouts to actionable process parameters and product-relevant quality attributes. Targeted assays require internal standards, calibration strategies, system suitability controls, and defined performance characteristics such as specificity, precision, linearity, sensitivity, and robustness. Untargeted workflows require pooled QC samples, drift correction, batch-effect management, and independent validation before candidate markers can guide manufacturing decisions. Ultimately, MS-based PAT will be most useful when connected to manufacturing outcomes such as expansion rate, viability, phenotype, potency, and post-thaw recovery.

## 6. Final Drug Product Characterization

Final drug product characterization is the point at which variability introduced across donor selection, genome editing, activation, expansion, harvest, formulation, and cryopreservation converges into the allogeneic CAR-T product administered to patients. Routine lot release remains anchored in targeted assays for identity, purity, safety, viability or strength, vector copy number, sterility, mycoplasma, endotoxin, and potency. FDA guidance specifically recommends identity testing that captures both the CAR transgene and intended cellular composition, requires potency testing of the final CAR-T drug product, and identifies vector copy number as an important safety attribute for integrating vector systems [20]. For allogeneic CAR-T lots intended to treat multiple patients, FDA further notes that additional adventitious-agent testing, stringent limits on potentially alloreactive lymphocytes, and absence of aberrant growth may be appropriate [20].

However, final release assays are not designed to fully resolve the molecular state of a living cellular product. This limitation becomes especially important during comparability assessment, where manufacturing changes may affect product biology without producing obvious changes in standard release results. FDA guidance states that meeting current lot release criteria is typically insufficient to establish comparability, and EMA guidance similarly frames release testing as a starting point that should be supplemented by extended characterization assays, especially for functional and biological product attributes [20,127]. MS is therefore best positioned as an orthogonal characterization platform for CQA discovery, comparability, process development, and failed-lot investigation rather than as a near-term replacement for routine release testing [17,128].

### 6.1. Proteomic and Phosphoproteomic Profiling of Final Product State

Global LC–MS/MS proteomics can provide a broad molecular readout of final CAR-T product state by quantifying proteins involved in activation, cytotoxicity, cytokine signaling, exhaustion, apoptosis, stress response, metabolism, and mitochondrial function [17,29,128,129]. This is valuable because two lots may show similar CAR expression, viability, and basic cytotoxicity while differing in cytotoxic effector abundance, stress-response pathways, metabolic enzyme expression, or exhaustion-associated protein networks. CAR-T proteomic and immunoproteomic studies support this rationale by showing that CAR constructs and intracellular signaling domains can generate distinct signaling complexes and protein-network states [29,129].

Phosphoproteomics adds a more direct readout of signaling activity by measuring phosphorylation-dependent pathways downstream of CAR engagement, costimulatory signaling, cytokine receptors, PI3K–AKT–mTOR, MAPK, and stress-response pathways [130]. Because phosphoproteomics is enrichment-intensive and more technically demanding than global proteomics, it is best suited for mechanistic characterization, construct comparison, comparability studies, and atypical-lot investigation. More mature translation would likely come from targeted PRM/SRM or locked DIA panels for predefined proteins or phosphopeptides identified during discovery [130,131].

### 6.2. MS-Enabled Single-Cell Profiling of Product Heterogeneity

Final CAR-T products contain heterogeneous mixtures of cells with distinct differentiation, activation, cytotoxic, exhausted, proliferative, and stress-associated states. Mass cytometry, or CyTOF, addresses this by using heavy-metal isotope-tagged antibodies to quantify dozens of predefined markers at single-cell resolution [67]. Unlike untargeted LC–MS proteomics, CyTOF is antibody-panel-dependent, but it is highly useful for resolving whether a product contains shifted proportions of naïve-like, memory-like, effector, exhausted, NK-like, activated, or senescent subpopulations. Single-cell and mass-cytometry studies in CAR-T therapy support the relevance of this approach. A donor-derived CD19 CAR-T study used single-cell multi-omics and mass cytometry to characterize pre- and post-infusion CAR+ and CAR− T cells, identifying heterogeneous differentiation trajectories and NK-like subsets associated with clinical outcomes [132]. Recent CAR-T atlas studies further support the concept that rare pre-infusion or persistent product states can be linked to durable remission [133]. For allogeneic CAR-T DP characterization, CyTOF is therefore most useful for development-stage heterogeneity mapping and comparability support, not routine release replacement.

### 6.3. Metabolomics and Lipidomics for Immunometabolic Fitness

Metabolomics and lipidomics provide functional information about final CAR-T quality because T-cell activity depends on mitochondrial fitness, redox capacity, nutrient utilization, biosynthetic readiness, and membrane remodeling (Table 5). Pre-infusion CD19 CAR-T products from long-term responders showed increased oxidative phosphorylation, fatty-acid oxidation, pentose phosphate pathway activity, mitochondrial mass, tighter cristae, and lower mTOR expression compared with products from short-term responders [134]. This suggests that metabolic state can encode clinically relevant product information not captured by conventional release assays.

Lipidomics adds information about membrane composition, sphingolipid biology, lipid utilization, lipid-raft organization, and stress-associated membrane remodeling. Broader T-cell studies show that activation and differentiation involve coordinated changes in metabolites, fatty acids, phospholipids, glycerolipids, and sphingolipids [63,64]. For allogeneic final drug products, these structural profiles serve as essential comparative blueprints, establishing deep structural equivalence whenever a process transitions across different scales, media lots, or bioreactor designs (Table 5).

### 6.4. Cryopreservation-Associated Stress and Post-Thaw Quality

Cryopreservation is central to allogeneic CAR-T manufacturing because centralized production, storage, shipping, and multi-patient distribution generally require a frozen final product. FDA notes that cryopreservation provides time for full release testing and logistical flexibility, but also that cryoprotectant risks and controlled thawing should be assessed because thawing can affect product quality [20]. Standard viability assays remain essential, but they may not fully capture delayed apoptosis, mitochondrial dysfunction, oxidative stress, membrane injury, or impaired post-thaw recovery.

MS-based proteomics, metabolomics, and lipidomics can provide deeper characterization of cryopreservation stress. Proteomics can identify stress-response, apoptosis, cell-cycle, cytoskeletal, and mitochondrial pathway shifts; metabolomics can detect altered energy metabolism, redox imbalance, and mitochondrial dysfunction; and lipidomics can reveal membrane remodeling, phospholipid degradation, ceramide accumulation, or sphingolipid changes [135,136]. These assays are particularly relevant for formulation development, cryoprotectant comparison, shipping-condition assessment, and comparability after changes in freezing or thawing protocols.

### 6.5. Process-Related Impurity and Residual Reagent Profiling

Final CAR-T drug product quality also depends on clearance or control of process-related impurities and residual reagents, including activation reagents, cytokines, media components, genome-editing reagents, nucleases, guide RNAs, plasmids, viral or nonviral delivery components, feeder- or matrix-derived proteins, serum proteins, purification reagents, and formulation excipients. Conventional ELISA-based impurity assays can be sensitive and practical when appropriate antibodies exist, but they often provide aggregate signals and may not distinguish individual residual protein species.

MS can provide orthogonal molecular specificity by identifying and quantifying individual process-related impurities [137]. This principle is well established in host-cell protein analysis for biologics, where LC–MS can complement ELISA by revealing which residual proteins are present rather than only measuring total immunoreactivity [137]. For allogeneic CAR-T, impurity MS is most defensible as a risk-based development and comparability tool: broad profiling can identify product-specific residual species, and selected sentinel impurities can later be translated into targeted MS or conventional assays.

### 6.6. Translation into Targeted CQA and Surrogate Assay Strategies

The main translational challenge is converting high-dimensional MS data into actionable product knowledge. Discovery features from proteomics, phosphoproteomics, metabolomics, lipidomics, or CyTOF should not be treated as CQAs unless they are reproducible, analytically robust, linked to product function or safety, and interpretable within a defined manufacturing context (Table 5). A practical workflow is to pair MS characterization with conventional release results, in-process data, post-thaw recovery, functional potency assays, and available in vivo or clinical correlates.

A critical consideration in advancing MS for CAR-T manufacturing is how these data correlate with established gold-standard methods, such as flow cytometry for phenotyping and ELISA for cytokine profiling. Currently, MS rarely serves as a direct surrogate for these traditional targeted readouts; rather, it provides complementary, orthogonal information. While flow cytometry excels at rapidly quantifying predefined cellular subsets, it cannot capture the dynamic intracellular metabolic or signaling networks that ultimately drive T cell activation and those subsequent surface phenotypes. Similarly, while targeted MS can quantify secreted cytokines, standard immunoassays like ELISA remain superior for routine, high-sensitivity release testing. Where MS adds indispensable value is in providing antibody-independent multiplexing and molecular specificity—identifying exact structural variants, proteoforms, post-translational modifications, and uncharacterized process-related impurities that conventional assays are inherently blind to, as ELISA typically only detects the bulk ensemble of immunoreactive molecules. Therefore, MS is best positioned not as an immediate replacement for standard readouts, but as a deeper orthogonal layer that contextualizes them during process development, comparability studies, and multi-attribute monitoring.

The most realistic endpoint is not routine deployment of broad discovery omics, but the development of focused, fit-for-purpose targeted assay panels. For LC–MS-based product characterization, this could take the form of a multi-attribute method or MAM-like workflow [138,139] that monitors predefined protein [19,78], phosphopeptide [131], metabolite, lipid [140,141], or sentinel impurity markers. Fixed CyTOF panels may provide an analogous targeted single-cell phenotyping layer [68]. By prioritizing validated, pre-selected attributes rather than broad discovery features, these assays could be applied more realistically to comparability studies, stability tracking, atypical-lot investigation, and release-adjacent characterization workflows. FDA and EMA guidance are consistent with this staged approach: early characterization supports CQA identification, while methods used for comparability or release must be sufficiently qualified, robust, and sensitive [10,127].

### 6.7. Current Limitations and Outlook

MS-based DP characterization still faces important limitations. Direct evidence in allogeneic CAR-T final drug products remains limited, with much of the strongest mechanistic evidence coming from autologous CAR-T products, donor-derived clinical studies, broader T-cell biology, or adoptive-cell-therapy analytical development studies. In addition, most LC–MS workflows are destructive and sensitive to cell input, sampling time, washing, quenching, extraction, digestion, storage, freeze–thaw history, instrument drift, and batch structure.

Despite these limitations, MS is well aligned with the analytical needs of allogeneic CAR-T development. Untargeted MS can define molecular mechanisms and candidate CQAs; CyTOF can resolve product heterogeneity; targeted MS can translate selected features into robust assays; and impurity MS can provide molecular specificity where immunoassays are limited. The most defensible role for MS is therefore not to replace standard release testing, but to make final drug product quality more mechanistically interpretable and to support the development of targeted next-generation comparability, potency, stability, and impurity-control strategies.

## 7. Post-Infusion Biomarker and Translational Monitoring

Post-infusion monitoring of CAR-T therapies is currently driven by clinical assessment, routine laboratory testing, cytokine measurements, flow cytometry, and molecular assays for CAR-T expansion and persistence. Foundational studies in CD19 CAR-T have shown that severe cytokine release syndrome (CRS) and immune effector cell-associated neurotoxicity syndrome (ICANS) are associated with inflammatory, myeloid, endothelial, and blood–brain barrier-related biomarkers, including IL-6, IFN-γ, MCP-1/CCL2, angiopoietin-2, von Willebrand factor, ferritin, and other inflammatory mediators [142,143,144,145]. However, these studies were largely based on cytokine panels, immunoassays, clinical laboratory measurements, and cellular assays rather than mass spectrometry. Therefore, post-infusion MS should be framed as an emerging translational layer rather than an established clinical monitoring standard for CAR-T.

Within this translational role, MS can help connect product attributes, manufacturing history, and patient-level molecular response after infusion. Plasma or serum LC–MS/MS proteomics may be used to profile inflammatory, complement, coagulation, acute-phase, endothelial, and tissue-injury proteins, while metabolomics and lipidomics may capture systemic metabolic stress, mitochondrial dysfunction, redox imbalance, steroid exposure, or organ injury. These applications are especially relevant for allogeneic CAR-T products, where post-infusion outcomes reflect interactions among donor-derived CAR-T cells, host immunity, lymphodepletion, tumor burden, and rescue interventions such as corticosteroids or IL-6 pathway blockade. The most practical workflow is a staged one: discovery-oriented LC–MS/MS, particularly data-independent acquisition proteomics, can nominate candidate toxicity or response signatures, but clinically meaningful translation would require reduction to focused PRM, SRM/MRM, or immuno-enrichment MS assays with stable-isotope standards [57,146,147].

The major barrier to post-infusion MS adoption is not the availability of instrumentation, but the need for carefully controlled clinical sampling, validation, and context-of-use definition. Post-infusion samples are vulnerable to anticoagulant choice, processing delay, hemolysis, platelet contamination, freeze–thaw history, infection status, timing relative to lymphodepletion, and exposure to steroids, tocilizumab, anakinra, or other interventions; plasma-proteomics studies have shown that such preanalytical variables can generate biomarker-like artifacts [48,148]. For allogeneic CAR-T, the most defensible near-term role of MS is therefore in well-annotated translational studies with baseline, pre-infusion, early toxicity-window, event-triggered, and later immune-reconstitution or relapse timepoints. Any MS assay intended to guide clinical care would require a defined biomarker context of use, analytical validation, locked quality-control criteria, and implementation in an appropriately regulated clinical laboratory environment [149,150]. Thus, MS should be presented as a promising bridge between CAR-T product characterization and patient response biology, while validated post-infusion MS biomarkers remain an area for further clinical development.

## 8. Conclusions

Across the allogeneic CAR-T workflow, mass spectrometry serves two distinct translational roles. For upstream gene-editing components, MS is increasingly utilized as a formal GMP release method—specifically for identity testing via intact sgRNA analysis, sequence variant screening, and critical attribute quantification such as 5′Cap mapping and poly(A) tail distribution. Conversely, for cellular in-process monitoring and final drug product characterization, MS serves primarily as an orthogonal platform for process development, CQA identification, comparability assessment, and impurity control. While operational constraints—such as destructive sampling, high cell inputs, and turnaround times—currently limit routine cell-product MS release testing, targeted MS panels (MAM, PRM) offer a realistic bridge to support comparability and lifecycle management in cell therapy manufacturing.

Allogeneic CAR-T manufacturing introduces a distinctive analytical challenge: a single donor-derived, extensively engineered, expanded, and cryopreserved cell product must be manufactured with sufficient consistency to support treatment of multiple patients. Across this workflow, conventional assays remain essential for donor eligibility, identity, purity, safety, viability, vector copy number, sterility, and potency testing. However, these assays are often targeted, endpoint-based, and limited in their ability to resolve the broader molecular states that shape manufacturing performance and final product quality. Mass spectrometry provides a complementary analytical layer by enabling deeper characterization of donor and leukapheresis material, genome-editing reagents, culture-media dynamics, cellular proteomic and metabolic states, lipid remodeling, process-related impurities, and post-thaw product quality. In process development and extended downstream product characterization, MS is most valuable as a stage-specific tool for process understanding, CQA discovery, comparability assessment, impurity investigation, and development of targeted surrogate assays.

The future impact of MS in allogeneic CAR-T manufacturing will depend on translation from broad discovery workflows into robust, fit-for-purpose methods that are linked to actionable manufacturing and product-quality endpoints. Global proteomics, metabolomics, lipidomics, phosphoproteomics, and MS-enabled single-cell approaches can identify candidate molecular features associated with donor variability, editing-material quality, expansion trajectory, cryopreservation stress, and product function. Yet these features should only be advanced toward routine use when they are reproducible, analytically controlled, and associated with defined outcomes such as editing efficiency, expansion kinetics, phenotype, potency, impurity clearance, stability, or post-thaw recovery. As targeted MS panels, at-line media-monitoring platforms, impurity assays, and multivariate process models mature, MS has the potential to strengthen quality-by-design strategies for allogeneic CAR-T products. Ultimately, its greatest contribution will be to make cell therapy manufacturing more mechanistically interpretable, enabling more rational control of variability across the donor-to-drug-product continuum.

Beyond analytical challenges, the integration of MS into routine CAR-T manufacturing is currently constrained by significant logistical and operational barriers. High-resolution mass spectrometers require substantial initial capital investment and costly ongoing maintenance, which can be prohibitive for decentralized or early-stage manufacturing facilities. Furthermore, traditional MS workflows—particularly global proteomics and native MS—suffer from prolonged turnaround times due to labor-intensive sample preparation, lengthy chromatographic separations, and complex computational data processing. These multi-day timelines are often incompatible with the rapid decision-making required for in-process monitoring or the timely release of cryopreserved cell products. Finally, operating these platforms and interpreting high-dimensional multi-omics data still demands a high degree of specialized, hands-on expertise. Until automated sample preparation, user-friendly bioinformatics pipelines, and simplified benchtop MS analyzers become universally adopted, the reliance on specialized personnel remains a primary bottleneck to the widespread implementation of MS in cell therapy quality control.

While this review focuses on the multi-stage manufacturing of allogeneic CAR-T cells, the mass spectrometry modalities, multi-attribute methods (MAM), and process analytical tools detailed here provide a universal framework for the broader adoptive cell therapy field. Key analytical layers—including intact oligonucleotide mapping for gene-editing reagents, at-line spent-media profiling, host-cell protein impurity characterization, and immunometabolic fitness monitoring—are directly transferable to autologous CAR-T, TCR-T, CAR-NK, and stem cell-derived therapies. As these advanced cell therapy platforms mature, stage-specific MS analytics will play an increasingly vital role in establishing process comparability and ensuring product safety and consistency across all engineered cell modalities.

## Figures and Tables

**Table 1:** MS-based and complementary analytical approaches for donor and cellular starting-material characterization in allogeneic CAR-T manufacturing.

Analytical Layer	Representative Method	Sample Type	Main Readout	Potential CAR-T Manufacturing Relevance	Maturity
Cellular proteomics	DDA/DIA LC–MS/MS	PBMCs or enriched T cells	Protein programs related to activation, stress response, memory state, metabolism, apoptosis, and cytotoxicity	Identifies donor-associated immune-cell programs that may correlate with activation response, transduction/editing efficiency, expansion kinetics, phenotype, potency, or post-thaw recovery	Discovery/process development
Multiplexed cellular proteomics	TMT or other isobaric labeling	PBMCs or enriched T cells	Relative protein abundance across donor cohorts or process conditions	Enables efficient donor-to-donor comparison, pathway discovery, and controlled multiplexed process-development studies	Discovery/process development
Targeted proteomics	MRM/SRM, PRM, triggered PRM	PBMCs, enriched T cells, plasma, or serum	Predefined protein or peptide markers	Monitors verified donor or starting-material markers; may support donor comparability or material-attribute panels after validation	Translational/fit-for-purpose after validation
Plasma proteomics	DIA, targeted plasma proteomics, depletion/enrichment, nanoparticle-corona workflows	Plasma or serum	Inflammatory, complement, acute-phase, coagulation, and metabolic-associated proteins	Identifies systemic donor-state covariates that may contextualize cellular phenotype or manufacturing performance	Discovery/translational
Cellular or plasma metabolomics	Targeted LC–MS metabolomics	PBMC extracts, enriched T-cell extracts, plasma, or serum	Amino acids, nucleotides, acylcarnitines, redox metabolites, and energy-related metabolites	Captures immunometabolic state potentially linked to activation, expansion, viability, differentiation, or stress tolerance	Translational/process development
Untargeted metabolomics	LC–MS or GC–MS metabolomics	PBMCs, enriched T cells, plasma, or serum	Broad metabolic feature maps	Supports hypothesis generation and discovery of candidate metabolic material attributes	Discovery
Lipidomics	LC–MS/MS or LC–HRMS lipidomics	PBMCs, enriched T cells, plasma, or serum	Lipid classes, sphingolipids, phospholipids, acyl-chain composition, and lipid-remodeling signatures	May capture membrane remodeling, lipid-raft biology, signaling state, and differentiation-associated lipid programs	Discovery/process development
Fluxomics	Stable-isotope tracing LC–MS	Ex vivo PBMCs or enriched T cells	Pathway activity and carbon/nitrogen flux	Provides mechanistic assessment of metabolic pathways supporting activation, proliferation, memory maintenance, or stress adaptation	Mechanistic research
MS-enabled immune phenotyping	CyTOF/mass cytometry	PBMCs or enriched T cells	Predefined immune markers at single-cell resolution	Quantifies donor-cell subset composition, memory/effector balance, activation, senescence, exhaustion, and myeloid/accessory-cell populations	Translational; complementary to MS omics
Emerging single-cell proteomics	nanoLC–MS single-cell proteomics	Individual cells	Endogenous proteins in single cells	Resolves proteomic heterogeneity masked by bulk profiling, but currently limited by throughput, robustness, and missingness	Emerging research
Complementary functional phenotyping	Single cell secretome chips	Individual cells or microchambers	Cytokine secretion and polyfunctionality	Provides functional context for donor-cell heterogeneity; useful alongside MS but not conventional LC–MS	Complementary/non-MS
Spatial immune profiling	IMC, MIBI-TOF	Tissue sections	Spatially resolved immune phenotypes	More relevant for tumor microenvironment or post-infusion translational studies than routine leukapheresis screening	Translational/research

**Abbreviations:** DDA, data-dependent acquisition; DIA, data-independent acquisition; TMT, tandem mass tag; MRM, multiple reaction monitoring; SRM, selected reaction monitoring; PRM, parallel reaction monitoring; CyTOF, cytometry by time-of-flight; IMC, imaging mass cytometry; MIBI-TOF, multiplexed ion beam imaging by time-of-flight. Table note: CyTOF, IMC, and MIBI-TOF are MS-enabled phenotyping platforms, whereas single-cell secretome chips are complementary functional assays rather than conventional LC–MS workflow2.2 Analytical Implementation and matrix-specific sample preparation.

**Table 2:** **Matrix-specific sample preparation and analytical implementation considerations for donor-stage MS workflows.** This table summarizes practical implementation considerations for donor and leukapheresis-stage MS analyses, including typical preparation steps, matrix-specific analytical vulnerabilities, and common mitigation strategies.

Matrix/Workflow	Typical Preparation	Main Analytical Issue	Common Mitigation Strategy	Best Use
PBMC or enriched T-cell proteomics	Lysis, digestion, SP3/S-Trap, or desalting	Detergent/salt interference; low input; digestion variability	SP3/S-Trap clean-up; internal standards; pooled QC	Discovery proteomics; donor comparability
Plasma/serum proteomics	Depletion, fractionation, or nanoparticle-corona enrichment	Extreme dynamic range; albumin/IgG masking low-abundance proteins	Targeted depletion/enrichment; stable isotope standards	Systemic inflammatory, coagulation, and covariate profiling
Targeted protein panels	Proteotypic peptide selection; heavy isotope standards	Peptide interference; matrix effects; stringent validation requirements	Optimization of transitions; rigorous calibration (LOD/LOQ)	Candidate donor-material attribute quantification
Single-cell proteomics	Single-cell isolation; miniaturized lysis/digestion	Severe input limitations; data missingness; technical variability	Miniaturized sample prep; carrier designs; high-sensitivity MS	Emerging discovery of rare donor-cell populations
Cellular metabolomics	Rapid quench; cold solvent extraction	Rapid ex vivo metabolite turnover; enzymatic degradation	Fast processing; cold solvents; batch correction; pooled QC	Immunometabolic and cell-activation state profiling
Plasma/serum metabolomics	Protein precipitation, SPE, or solvent extraction	Pre-analytical variables (diet, tube type, freeze–thaw); matrix effects	Standardized collection protocols; pooled QC correction	Systemic metabolic covariate profiling
Lipidomics	Organic or biphasic extraction	Isomeric ambiguity; polar/nonpolar coverage bias	Biphasic extraction; class-specific internal standards	Membrane remodeling; lipid-raft and signaling studies
Fluxomics	Ex vivo isotope labeling; rapid quench; isotopologue analysis	Low throughput; complex interpretation; labeling design sensitivity; isotope natural-abundance correction	Prespecified labeling design; algorithmic pathway modeling	Mechanistic pathway studies, not routine donor screening
Mass cytometry/CyTOF	Metal-conjugated antibody staining; single-cell nebulization; ICP ionization; TOF-MS	Antibody-panel dependence; batch effects; limited to predefined markers; lower throughput	Barcoding; standardized panels; reference controls; orthogonal flow confirmation	High-dimensional donor immunophenotyping and subpopulation mapping
Single-cell LC–MS proteomics	Single-cell isolation; miniaturized lysis/digestion; low-flow LC; high-sensitivity MS	Severe input limitations; data missingness; technical variability; low throughput	Miniaturized prep; carrier/reference designs; low-flow LC; strict QC	Emerging discovery of rare donor-cell states
Imaging mass cytometry/MIBI-TOF	Metal-tagged antibody staining of tissue; laser ablation or ion-beam imaging; mass-based readout	Image segmentation and spatial analysis burden; limited direct relevance to leukapheresis	Tissue-specific panels; segmentation QC; spatial analysis pipelines; orthogonal IHC	Tumor microenvironment and tissue-context immune profiling

**Abbreviations:** SPE, solid-phase extractionHRMS, high-resolution mass spectrometry; LOD, limit of detection; LOQ, limit of quantification; ICP, inductively coupled plasma; TOF-MS, time-of-flight mass spectrometry; QC, quality control.

**Table 3:** **Critical Evaluation of Mass Spectrometry Paradigms for Genome Editing Starting Materials.**

Starting Material	MS Analytical Workflow	Primary Quality Attributes Addressed	Key Technical Advantages	Current Technical Bottlenecks & Operational Constraints
sgRNA/mRNA	Intact and Top-Down HRMS	Molecular weight confirmation; major modification profiling; N+1; N-1 impurities.	Preserves intact sequence context; avoids sample preparation or digestion artifacts.	Spectral congestion and charge envelope overlap for sequences > 100 nt; requires advanced gas-phase deconvolution (cIMS/SLIM).
sgRNA/mRNA	Bottom-Up Mapping (Enzymatic Digestion)	Sequence verification; SVA; low-abundance point mutations.	High sensitivity; localizes positional isomers; detects trace impurities down to 0.1%	Labor-intensive multi-enzymatic steps; risk of losing macrostructural alignment/long-range connectivity context.
sgRNA/mRNA	nMS	Non-covalent multimeric aggregates; immunogenic 3′-loopback dsRNA impurities.	Preserves native folding states intact non-covalent double strands.	Online coupling is technically challenging; raw sample salt removal requires automated descaling/buffer-exchange. native workflows face lower operational throughput.
mRNA Termini	Targeted Clipping, Bottom-Up Mapping	Capping efficiency; 3′ Poly(A) tail distribution;	Achieves single-nucleotide resolution mapping of large terminal regions	Requires transcript-specific DNAzyme/RNase H design
Nucleases/RNPs	nMS and IM-MS	HOS; multi-domain folding; RNP stoichiometry; assembly efficiency.	Preserves non-covalent interactions; resolves true macromolecular complexes under physiological states.	Requires volatile, near-neutral pH buffer optimization; automated data deconvolution software remains a major bottleneck.
CAR Transgene/dsDNA	Restriction Digest & CD-MS	Sequence fidelity; modification mapping; supercoiled vs. linear plasmid topology.	Definitive sequence-level auditing combined with single-particle macrostructural folding analytics.	High molecular size limits direct analysis: computational charging data interpretation requires specialized expertise.

Abbreviation: CD-MS: Charge Detection Mass Spectrometry; cIMS: cyclic ion mobility spectrometry; dsDNA: Double-Stranded Deoxyribonucleic Acid; dsRNA: Double-Stranded Ribonucleic Acid; HRMS: high-resolution mass spectrometry; IM-MS: ion mobility mass spectrometry; mRNA: Messenger Ribonucleic Acid; nMS: native mass spectrometry; nt: nucleotides; RNP: ribonucleoprotein; sgRNA: Single-Guide Ribonucleic Acid; SLIM: structures for lossless ion manipulation; SVA: Sequence Variant Analysis.

**Table 4:** **Analytical technologies for MS-based and MS-adjacent in-process monitoring during CAR T-cell expansion.** Technologies are organized by likely implementation tier. Targeted spent-media analysis and online glucose/lactate monitoring are the most practical near-term PAT approaches, whereas untargeted metabolomics, fluxomics, secretome proteomics, surfaceomics, cellular proteomics, and lipidomics are currently better positioned for process development, mechanistic studies, comparability assessment, and candidate CQA discovery.

Application Tier	Technology/Modality	Sample Type	Representative Readouts	Potential Use During CAR T-Cell Expansion	Representative Evidence/Examples	Readiness and Limitations
Near-term PAT/operational monitoring	Online glucose/lactate monitoring	Bioreactor media, online sampling	Glucose depletion; lactate accumulation	Guides feed adjustment, perfusion rates, and prevents nutrient exhaustion	MAVEN system; linking dynamics to yield/viability	High operational relevance; complementary to MS but limited in analyte scope.
Near-term PAT/at-line monitoring	Targeted spent-media nutrient analysis by CE–MS	Small-volume fresh or spent media	Amino acids, vitamins, choline, selected components	Tracks nutrient depletion to guide feeding, media exchange, harvest, or process drift	REBEL/REBEL XT targeted media analyzers	Most viable near-term MS PAT; utilizes fixed panels; requires process qualification.
Process development/media optimization	Untargeted extracellular metabolomics	Spent media	Broad nutrient consumption and metabolite accumulation	Identifies metabolic signatures of expansion kinetics, exhaustion, or process variability	LC–MS, GC–MS, or NMR studies of activated T-cell metabolism	Discovery-oriented; annotation challenges and throughput limits restrict routine PAT use.
Process development/mechanistic biology	Intracellular metabolomics	T-cell pellets	Glycolysis/TCA intermediates, nucleotides, redox state	Characterizes intracellular metabolic state during activation, proliferation, and stress	CD8+ T-cell activation and LC-MS or GC-MS studies of activated T-cell metabolomics	Destructive sampling (requires extraction); ideal for development, not real-time control.
Mechanism discovery/media design	Stable-isotope tracing/fluxomics	Labeled cells and/or spent media	Carbon/nitrogen routing through metabolic pathways	Evaluates how specific media formulations or conditions alter nutrient utilization	13C-glucose and isotope-tracing studies in T-cells	Mechanistically powerful but expensive, destructive, and low-throughput; unsuitable for PAT.
Functional-state characterization	Targeted secretome proteomics by MRM/SRM/PRM	Culture supernatant	Cytokines, chemokines, stress/damage markers	Complements ELISA or bead assays for multiplexed immune and stress monitoring	MRM/SRM cytokine quantification in primary cells	Useful for validated panels; limited by sensitivity, matrix effects, and turnaround time.
Process characterization/CQA discovery	Cell-surface proteomics/surfaceomics	Live-cell labeled T cells	Surface glycoproteins, nutrient transporters, adhesion/homing receptors	Identifies surface programs linked to memory, activation, exhaustion, or hypoxia	Cell-surface capture glycoproteomics of CD8+ T-cells	Strong for discovery/comparability; requires cell harvest, labeling, and longer analysis time.
Process characterization/CQA discovery	Global cellular proteomics	T-cell pellets	Signaling proteins, metabolic enzymes, stress/apoptosis proteins	Characterizes intracellular programs linked to expansion performance, potency, or phenotype	Quantitative immune-cell and T-cell proteomics	High biological depth but destructive and analytically complex; ideal for development.
Membrane-state and persistence biology	Global lipidomics	T-cell pellets or membrane fractions	Cholesterol, phospholipids, sphingolipids, free fatty acids	Evaluates lipid remodeling tied to activation, persistence, stress adaptation, and lipid rafts	T-cell lipid storage, stress adaptation, and glycosphingolipid studies	Valuable for mechanism discovery; requires careful extraction, annotation, and batch control.
Future PAT/model-based control	Soft sensors & multivariate models	Integrated process data streams	Predicted nutrient demand, biomass, harvest readiness, drift risk	Converts sparse metabolite and process measurements into actionable control signals	PAT framework; adaptive perfusion and metabolic-control concepts	Promising, but demands strict model validation, lifecycle management, and CQA linkage.
Emerging/future-facing	Miniaturized or automated MS-coupled culture systems	Microbioreactor samples, automated at-line samples	Media metabolites, potentially proteins or lipid markers depending on integration	Could enable smaller volume, more frequent sampling and decentralized process monitoring	Microfluidic CAR T manufacturing platforms and automated sampling concepts	Not yet routine. Challenges include aseptic integration, fouling, GMP software, calibration stability, and data integrity.

**Table 5:** **Emerging MS-Derived Molecular Profiles, Associated Functional Outcomes, and Product Quality Impacts.**

Omics Category	Specific MS-Derived Signature/Pathway	Associated Functional/Biological Outcome	Potential Product Quality Impact
Metabolomics	Elevated Oxidative Phosphorylation (OXPHOS), Fatty Acid Oxidation, Pentose Phosphate Pathway	Enhanced mitochondrial fitness, memory-like phenotype maintenance	High pre-infusion persistence, long-term clinical response
Lipidomics	Glucose-dependent glycosphingolipid biosynthesis upregulation	Improved lipid-raft aggregation following TCR stimulation	Enhanced CD8+ T-cell expansion, cytotoxicity, and tumor control
Lipidomics	Reduced Acetyl-CoA carboxylase (ACC) activity/limited lipid storage	Preserved cellular energy balance under metabolic stress	Sustained polyfunctionality and persistence in immunosuppressive environments
Surface Proteomics	Remodeled cell-surface glycoproteome & altered nutrient transporters under hypoxia	Adaptation to low-oxygen/nutrient-deprived microenvironments	Early marker of metabolic stress preceding T-cell exhaustion
Phosphoproteomics	Differential CAR costimulatory domain phosphorylation kinetics (e.g., CD28 vs. 4-1BB)	Distinct signaling activation thresholds and downstream metabolic wiring	Informs construct selection, signaling strength tuning, and functional persistence

## Data Availability

No new data were created or analyzed in this study. Data sharing is not applicable to this article.

## References

[1] U.S. Food and Drug Administration (FDA) August 30, 2017 Approval Letter-KYMRIAH. https://www.fda.gov/media/106989/download?attachment.

[2] U.S. Food and Drug Administration (FDA) October 18, 2017 Approval Letter-YESCARTA. https://www.fda.gov/media/108458/download?attachment.

[3] O’Leary M.C., Lu X., Huang Y., Lin X., Mahmood I., Przepiorka D., Gavin D., Lee S., Liu K., George B. (2019). FDA Approval Summary: Tisagenlecleucel for Treatment of Patients with Relapsed or Refractory B-Cell Precursor Acute Lymphoblastic Leukemia. Clin. Cancer Res..

[4] Depil S., Duchateau P., Grupp S.A., Mufti G., Poirot L. (2020). ‘Off-the-Shelf’ Allogeneic CAR T Cells: Development and Challenges. Nat. Rev. Drug Discov..

[5] Li Y.-R., Zhu Y., Fang Y., Lyu Z., Yang L. (2025). Emerging Trends in Clinical Allogeneic CAR Cell Therapy. Med.

[6] Cheema A.Y., Ali H.M., Maryam B., Aslam M.F., Thiagarajan P.S., Shahid D., Munir M., Najam A., Raza S., Anwer F. (2026). Under the Hood: Evidence-Based Review of Allogeneic Chimeric Antigen Receptor T Cells for Hematologic Malignancies. Transplant. Cell. Ther..

[7] Lyu Z., Niu S., Fang Y., Chen Y., Li Y.-R., Yang L. (2025). Addressing Graft-versus-Host Disease in Allogeneic Cell-Based Immunotherapy for Cancer. Exp. Hematol. Oncol..

[8] Su J., Zeng Y., Song Z., Liu Y., Ou K., Wu Y., Huang M., Li Y., Tu S. (2025). Genome-Edited Allogeneic CAR-T Cells: The next Generation of Cancer Immunotherapies. J. Hematol. Oncol..

[9] Mishra A.K., Qasim W. (2024). Genome Editing Approaches for Universal Chimeric Antigen Receptor T Cells. EJC Paediatr. Oncol..

[10] Jadlowsky J.K., Chang J., Spencer D.H., Warrington J.M., Levine B.L., June C.H., Fraietta J.A., Singh N. (2024). Regulatory Considerations for Genome-Edited T-Cell Therapies. Cancer Immunol. Res..

[11] U.S. Food and Drug Administration (FDA) (2024). Human Gene Therapy Products Incorporating Human Genome Editing: Guidance for Industry.

[12] U.S. Food and Drug Administration (FDA) (2025). Assessing Genetic Heterogeneity in the Context of Gene-Edited Products.

[13] Turicek D.P., Giordani V.M., Moraly J., Taylor N., Shah N.N. (2023). CAR T-Cell Detection Scoping Review: An Essential Biomarker in Critical Need of Standardization. J. Immunother. Cancer.

[14] Shao L., Zheng Y., Somerville R.P., Stroncek D.F., Jin P. (2025). New Insights on Potency Assays from Recent Advances and Discoveries in CAR T-Cell Therapy. Front. Immunol..

[15] Shoshi A., Xia Y., Fieschi A., Baumgarten Y., Gaißler A., Ackermann T., Reimann P., Mitschang B., Weyrich M., Bauernhansl T. (2025). An Analysis of Monitoring Solutions for CAR T Cell Production. Healthc. Technol. Lett..

[16] Foureau D., Bhutani M., Guo F., Rigby K., Leonidas M., Tjaden E., Fox A., Atrash S., Paul B., Voorhees P.M. (2021). Comparison of Mass Spectrometry and Flow Cytometry in Measuring Minimal Residual Disease in Multiple Myeloma. Cancer Med..

[17] Lombard-Banek C., Schiel J.E. (2020). Mass Spectrometry Advances and Perspectives for the Characterization of Emerging Adoptive Cell Therapies. Molecules.

[18] Lau H., Rogers R. (2021). Mass Spectrometry–Based Process Analytical Technologies for Cell Therapies. LCGC N. Am..

[19] Boja E.S., Fehniger T.E., Baker M.S., Marko-Varga G., Rodriguez H. (2014). Analytical Validation Considerations of Multiplex Mass-Spectrometry-Based Proteomic Platforms for Measuring Protein Biomarkers. J. Proteome Res..

[20] U.S. Food and Drug Administration (FDA) Considerations for the Development of Chimeric Antigen Receptor (CAR) T Cell Products: Guidance for Industry. https://www.fda.gov/regulatory-information/search-fda-guidance-documents/considerations-development-chimeric-antigen-receptor-car-t-cell-products.

[21] Campbell A., Brieva T., Raviv L., Rowley J., Niss K., Brandwein H., Oh S., Karnieli O. (2015). Concise Review: Process Development Considerations for Cell Therapy. Stem Cells Transl. Med..

[22] Cell and Gene Therapy Answers: Assessing the Critical Quality Attributes, Challenges and Importance of CMC. https://www.labcorp.com/education-events/articles/cell-and-gene-therapy-answers-assessing-critical-quality-attributes-challenges-and-importance-cmc.

[23] Levine B.L., Miskin J., Wonnacott K., Keir C. (2017). Global Manufacturing of CAR T Cell Therapy. Mol. Ther.-Methods Clin. Dev..

[24] Abou-el-Enein M., Elsallab M., Feldman S.A., Fesnak A.D., Heslop H.E., Marks P., Till B.G., Bauer G., Savoldo B. (2021). Scalable Manufacturing of CAR T Cells for Cancer Immunotherapy. Blood Cancer Discov..

[25] Ayala Ceja M., Khericha M., Harris C.M., Puig-Saus C., Chen Y.Y. (2024). CAR-T Cell Manufacturing: Major Process Parameters and next-Generation Strategies. J. Exp. Med..

[26] Marton C., Clémenceau B., Dachy G., Demerle C., Derenne S., Ferrand C., Giverne C., Latouche J.-B., Lemée L., Martinet J. (2025). Harmonisation of Quality Control Tests for Academic Production of CAR-T Cells: A Position Paper from the WP-Bioproduction of the UNITC Consortium. Bone Marrow Transpl..

[27] Roddie C., O’Reilly M., Dias Alves Pinto J., Vispute K., Lowdell M. (2019). Manufacturing Chimeric Antigen Receptor T Cells: Issues and Challenges. Cytotherapy.

[28] Qayed M., McGuirk J.P., Myers G.D., Parameswaran V., Waller E.K., Holman P., Rodrigues M., Clough L.F., Willert J. (2022). Leukapheresis Guidance and Best Practices for Optimal Chimeric Antigen Receptor T-Cell Manufacturing. Cytotherapy.

[29] Teibo J.O., Picanço-Castro V., De Souza L.E.B., Faça V.M. (2025). A Proteomics Outlook on the Molecular Effectors of CAR-T Cell Therapy in Cancer Management. J. Proteome Res..

[30] Eyquem J., Mansilla-Soto J., Giavridis T., van der Stegen S.J.C., Hamieh M., Cunanan K.M., Odak A., Gönen M., Sadelain M. (2017). Targeting a CAR to the TRAC Locus with CRISPR/Cas9 Enhances Tumour Rejection. Nature.

[31] Kawalekar O.U., O’Connor R.S., Fraietta J.A., Guo L., McGettigan S.E., Posey A.D., Patel P.R., Guedan S., Scholler J., Keith B. (2016). Distinct Signaling of Coreceptors Regulates Specific Metabolism Pathways and Impacts Memory Development in CAR T Cells. Immunity.

[32] Ghassemi S., Durgin J.S., Nunez-Cruz S., Patel J., Leferovich J., Pinzone M., Shen F., Cummins K.D., Plesa G., Cantu V.A. (2022). Rapid Manufacturing of Non-Activated Potent CAR T Cells. Nat. Biomed. Eng..

[33] Brudno J.N., Kochenderfer J.N. (2016). Toxicities of Chimeric Antigen Receptor T Cells: Recognition and Management. Blood.

[34] U.S. Food and Drug Administration (FDA) Cellular & Gene Therapy Guidances. https://www.fda.gov/vaccines-blood-biologics/biologics-guidances/cellular-gene-therapy-guidances.

[35] Song H.W., Prochazkova M., Shao L., Traynor R., Underwood S., Black M., Fellowes V., Shi R., Pouzolles M., Chou H.-C. (2024). CAR-T Cell Expansion Platforms Yield Distinct T Cell Differentiation States. Cytotherapy.

[36] Hood T., Slingsby F., Sandner V., Geis W., Schmidberger T., Bevan N., Vicard Q., Hengst J., Springuel P., Dianat N. (2024). A Quality-by-Design Approach to Improve Process Understanding and Optimise the Production and Quality of CAR-T Cells in Automated Stirred-Tank Bioreactors. Front. Immunol..

[37] Hood T., Springuel P., Slingsby F., Sandner V., Geis W., Schmidberger T., Bevan N., Vicard Q., Hengst J., Dianat N. (2025). Establishing a Scalable Perfusion Strategy for the Manufacture of CAR-T Cells in Stirred-tank Bioreactors Using a Quality-by-design Approach. Bioeng. Transla Med..

[38] Gostage J., Domingo-Lopez D.A., Tarpey R., Duffy G.P., Levey R.E. (2025). From Cold Chain to Ambient: Benefits, Risks, and Evidence across Cell Therapy Logistics. Mol. Ther. Methods Clin. Dev..

[39] Browne D.J., Miller C.M., Doolan D.L. (2024). Technical Pitfalls When Collecting, Cryopreserving, Thawing, and Stimulating Human T-Cells. Front. Immunol..

[40] U.S. Food and Drug Administration, Center for Biologics Evaluation and Research Eligibility Determination for Donors of Human Cells, Tissues, and Cellular and Tissue-Based Products. https://www.fda.gov/regulatory-information/search-fda-guidance-documents/eligibility-determination-donors-human-cells-tissues-and-cellular-and-tissue-based-products.

[41] Noaks E., Peticone C., Kotsopoulou E., Bracewell D.G. (2021). Enriching Leukapheresis Improves T Cell Activation and Transduction Efficiency during CAR T Processing. Mol. Ther. Methods Clin. Dev..

[42] Končarević S., Lößner C., Kuhn K., Prinz T., Pike I., Zucht H.-D. (2014). In-Depth Profiling of the Peripheral Blood Mononuclear Cells Proteome for Clinical Blood Proteomics. Int. J. Proteom..

[43] Rieckmann J.C., Geiger R., Hornburg D., Wolf T., Kveler K., Jarrossay D., Sallusto F., Shen-Orr S.S., Lanzavecchia A., Mann M. (2017). Social Network Architecture of Human Immune Cells Unveiled by Quantitative Proteomics. Nat. Immunol..

[44] Patti G.J., Yanes O., Siuzdak G. (2012). Metabolomics: The Apogee of the Omics Trilogy. Nat. Rev. Mol. Cell Biol..

[45] Blume J.E., Manning W.C., Troiano G., Hornburg D., Figa M., Hesterberg L., Platt T.L., Zhao X., Cuaresma R.A., Everley P.A. (2020). Rapid, Deep and Precise Profiling of the Plasma Proteome with Multi-Nanoparticle Protein Corona. Nat. Commun..

[46] Anderson N.L., Anderson N.G. (2002). The Human Plasma Proteome. Mol. Cell. Proteom..

[47] Geyer P.E., Kulak N.A., Pichler G., Holdt L.M., Teupser D., Mann M. (2016). Plasma Proteome Profiling to Assess Human Health and Disease. Cell Syst..

[48] Geyer P.E., Voytik E., Treit P.V., Doll S., Kleinhempel A., Niu L., Müller J.B., Buchholtz M., Bader J.M., Teupser D. (2019). Plasma Proteome Profiling to Detect and Avoid Sample-related Biases in Biomarker Studies. EMBO Mol. Med..

[49] Cao Z., Kamlage B., Wagner-Golbs A., Maisha M., Sun J., Schnackenberg L.K., Pence L., Schmitt T.C., Daniels J.R., Rogstad S. (2019). An Integrated Analysis of Metabolites, Peptides, and Inflammation Biomarkers for Assessment of Preanalytical Variability of Human Plasma. J. Proteome Res..

[50] Li H., Mao Y., Xiong Y., Zhao H.H., Shen F., Gao X., Yang P., Liu X., Fu D. (2019). A Comprehensive Proteome Analysis of Peripheral Blood Mononuclear Cells (PBMCs) to Identify Candidate Biomarkers of Pancreatic Cancer. Cancer Genom. Proteom..

[51] Haudek-Prinz V.J., Klepeisz P., Slany A., Griss J., Meshcheryakova A., Paulitschke V., Mitulovic G., Stöckl J., Gerner C. (2012). Proteome Signatures of Inflammatory Activated Primary Human Peripheral Blood Mononuclear Cells. J. Proteom..

[52] Hughes C.S., Moggridge S., Müller T., Sorensen P.H., Morin G.B., Krijgsveld J. (2019). Single-Pot, Solid-Phase-Enhanced Sample Preparation for Proteomics Experiments. Nat. Protoc..

[53] Zougman A., Selby P.J., Banks R.E. (2014). Suspension Trapping (STrap) Sample Preparation Method for Bottom-up Proteomics Analysis. Proteomics.

[54] Yin P., Peter A., Franken H., Zhao X., Neukamm S.S., Rosenbaum L., Lucio M., Zell A., Häring H.-U., Xu G. (2013). Preanalytical Aspects and Sample Quality Assessment in Metabolomics Studies of Human Blood. Clin. Chem..

[55] Vuckovic D. (2012). Current Trends and Challenges in Sample Preparation for Global Metabolomics Using Liquid Chromatography–Mass Spectrometry. Anal. Bioanal. Chem..

[56] Demichev V., Messner C.B., Vernardis S.I., Lilley K.S., Ralser M. (2020). DIA-NN: Neural Networks and Interference Correction Enable Deep Proteome Coverage in High Throughput. Nat. Methods.

[57] Gillet L.C., Navarro P., Tate S., Röst H., Selevsek N., Reiter L., Bonner R., Aebersold R. (2012). Targeted Data Extraction of the MS/MS Spectra Generated by Data-Independent Acquisition: A New Concept for Consistent and Accurate Proteome Analysis. Mol. Cell. Proteom..

[58] Thompson A., Schäfer J., Kuhn K., Kienle S., Schwarz J., Schmidt G., Neumann T., Hamon C. (2003). Tandem Mass Tags: A Novel Quantification Strategy for Comparative Analysis of Complex Protein Mixtures by MS/MS. Anal. Chem..

[59] Ting L., Rad R., Gygi S.P., Haas W. (2011). MS3 Eliminates Ratio Distortion in Isobaric Multiplexed Quantitative Proteomics. Nat. Methods.

[60] McAlister G.C., Nusinow D.P., Jedrychowski M.P., Wühr M., Huttlin E.L., Erickson B.K., Rad R., Haas W., Gygi S.P. (2014). MultiNotch MS3 Enables Accurate, Sensitive, and Multiplexed Detection of Differential Expression across Cancer Cell Line Proteomes. Anal. Chem..

[61] Buck M.D., O’Sullivan D., Pearce E.L. (2015). T Cell Metabolism Drives Immunity. J. Exp. Med..

[62] Edwards-Hicks J., Mitterer M., Pearce E.L., Buescher J.M. (2021). Metabolic Dynamics of In Vitro CD8+ T Cell Activation. Metabolites.

[63] Kanno T., Konno R., Sato M., Kurabayashi A., Miyako K., Nakajima T., Yokoyama S., Sasamoto S., Asou H.K., Ohzeki J. (2024). The Integration of Metabolic and Proteomic Data Uncovers an Augmentation of the Sphingolipid Biosynthesis Pathway during T-Cell Differentiation. Commun. Biol..

[64] Hunt E.G., Hurst K.E., Riesenberg B.P., Kennedy A.S., Gandy E.J., Andrews A.M., del Mar Alicea Pauneto C., Ball L.E., Wallace E.D., Gao P. (2024). Acetyl-CoA Carboxylase Obstructs CD8+ T Cell Lipid Utilization in the Tumor Microenvironment. Cell Metab..

[65] Longo J., DeCamp L.M., Oswald B.M., Teis R., Reyes-Oliveras A., Dahabieh M.S., Ellis A.E., Vincent M.P., Damico H., Gallik K.L. (2025). Glucose-Dependent Glycosphingolipid Biosynthesis Fuels CD8+ T Cell Function and Tumor Control. Cell Metab..

[66] Buescher J.M., Antoniewicz M.R., Boros L.G., Burgess S.C., Brunengraber H., Clish C.B., DeBerardinis R.J., Feron O., Frezza C., Ghesquiere B. (2015). A Roadmap for Interpreting 13C Metabolite Labeling Patterns from Cells. Curr. Opin. Biotechnol..

[67] Bendall S.C., Simonds E.F., Qiu P., Amir E.D., Krutzik P.O., Finck R., Bruggner R.V., Melamed R., Trejo A., Ornatsky O.I. (2011). Single-Cell Mass Cytometry of Differential Immune and Drug Responses Across a Human Hematopoietic Continuum. Science.

[68] Iyer A., Hamers A.A.J., Pillai A.B. (2022). CyTOF® for the Masses. Front. Immunol..

[69] Jang C., Chen L., Rabinowitz J.D. (2018). Metabolomics and Isotope Tracing. Cell.

[70] Michelozzi I.M., Sufi J., Adejumo T.A., Amrolia P.J., Tape C.J., Giustacchini A. (2022). High-Dimensional Functional Phenotyping of Preclinical Human CAR T Cells Using Mass Cytometry. STAR Protoc..

[71] Ma C., Fan R., Ahmad H., Shi Q., Comin-Anduix B., Chodon T., Koya R.C., Liu C.-C., Kwong G.A., Radu C.G. (2011). A Clinical Microchip for Evaluation of Single Immune Cells Reveals High Functional Heterogeneity in Phenotypically Similar T Cells. Nat. Med..

[72] Xue Q., Bettini E., Paczkowski P., Ng C., Kaiser A., McConnell T., Kodrasi O., Quigley M.F., Heath J., Fan R. (2017). Single-Cell Multiplexed Cytokine Profiling of CD19 CAR-T Cells Reveals a Diverse Landscape of Polyfunctional Antigen-Specific Response. J. Immunother. Cancer.

[73] Brunner A., Thielert M., Vasilopoulou C., Ammar C., Coscia F., Mund A., Hoerning O.B., Bache N., Apalategui A., Lubeck M. (2022). Ultra-high Sensitivity Mass Spectrometry Quantifies Single-cell Proteome Changes upon Perturbation. Mol. Syst. Biol..

[74] Budnik B., Levy E., Harmange G., Slavov N. (2018). SCoPE-MS: Mass Spectrometry of Single Mammalian Cells Quantifies Proteome Heterogeneity during Cell Differentiation. Genome Biol..

[75] Giesen C., Wang H.A.O., Schapiro D., Zivanovic N., Jacobs A., Hattendorf B., Schüffler P.J., Grolimund D., Buhmann J.M., Brandt S. (2014). Highly Multiplexed Imaging of Tumor Tissues with Subcellular Resolution by Mass Cytometry. Nat. Methods.

[76] Angelo M., Bendall S.C., Finck R., Hale M.B., Hitzman C., Borowsky A.D., Levenson R.M., Lowe J.B., Liu S.D., Zhao S. (2014). Multiplexed Ion Beam Imaging of Human Breast Tumors. Nat. Med..

[77] Kuzyk M.A., Smith D., Yang J., Cross T.J., Jackson A.M., Hardie D.B., Anderson N.L., Borchers C.H. (2009). Multiple Reaction Monitoring-Based, Multiplexed, Absolute Quantitation of 45 Proteins in Human Plasma. Mol. Cell. Proteom..

[78] Peterson A.C., Russell J.D., Bailey D.J., Westphall M.S., Coon J.J. (2012). Parallel Reaction Monitoring for High Resolution and High Mass Accuracy Quantitative, Targeted Proteomics. Mol. Cell. Proteom..

[79] Rauniyar N. (2015). Parallel Reaction Monitoring: A Targeted Experiment Performed Using High Resolution and High Mass Accuracy Mass Spectrometry. Int. J. Mol. Sci..

[80] Gallien S., Kim S.Y., Domon B. (2015). Large-Scale Targeted Proteomics Using Internal Standard Triggered-Parallel Reaction Monitoring (IS-PRM). Mol. Cell. Proteom..

[81] June C.H., Sadelain M. (2018). Chimeric Antigen Receptor Therapy. N. Engl. J. Med..

[82] Jinek M., Chylinski K., Fonfara I., Hauer M., Doudna J.A., Charpentier E. (2012). A Programmable Dual-RNA–Guided DNA Endonuclease in Adaptive Bacterial Immunity. Science.

[83] Gillmore J.D., Gane E., Taubel J., Kao J., Fontana M., Maitland M.L., Seitzer J., O’Connell D., Walsh K.R., Wood K. (2021). CRISPR-Cas9 In Vivo Gene Editing for Transthyretin Amyloidosis. N. Engl. J. Med..

[84] Auclair J., Rathore A.S. (2023). Analysis of Lipid Nanoparticles. LCGC Int..

[85] Schober G.B., Story S., Arya D.P. (2024). A Careful Look at Lipid Nanoparticle Characterization: Analysis of Benchmark Formulations for Encapsulation of RNA Cargo Size Gradient. Sci. Rep..

[86] Waters Corporation Characterization of Intact mRNA Using Ion Pair-Reversed Phase-Time of Flight-MS, Size Exclusion Chromatography-Multi Angle Light Scattering, and Charge Detection Mass Spectrometry. https://www.waters.com/nextgen/us/en/library/library-details.html?documentid=PSTR135098898&t=waters-CharacterizationofIntactmRNAUsingIonPairReversedPhaseTimeofFlightMS-PSTR135098898.

[87] Tutiš L., Ferguson P.D., Benstead D., Clarke A., Heatherington C., Gripton C., Vanhinsbergh C.J., Somsen G.W., Gargano A.F. (2025). Ion-Pairing Hydrophilic Interaction Chromatography for Impurity Profiling of Therapeutic Phosphorothioated Oligonucleotides. Anal. Chem..

[88] Crittenden C.M., Lanzillotti M.B., Chen B. (2023). Top-Down Mass Spectrometry of Synthetic Single Guide Ribonucleic Acids Enabled by Facile Sample Clean-Up. Anal. Chem..

[89] Macias L.A., Lowther J., Tillotson E.L., Rohde E., Madsen J.A. (2025). Ion Mobility Gas-Phase Separation Enhances Top-Down Mass Spectrometry of Heavily Modified Guide RNA. Anal. Chem..

[90] Blevins M.S., Du J., Aderorho R., Lieu R., Crittenden C.M., Chen T. (2026). High-Resolution Ion Mobility Mass Spectrometry for Separation of Oligonucleotide Phosphorothioate Diastereomers. Anal. Chem..

[91] Sharon E.M., O’Keefe S.M., Grassmyer K.T., Raab S.A., Maloney T.D., Clemmer D.E. (2024). Diastereomer Characterization of PS-Modified Synthetic Oligonucleotides Using Cyclic IMS-MS. Anal. Chem..

[92] O’Keefe S.M., Sharon E.M., Grassmyer K.T., Raab S.A., Maloney T.D., Clemmer D.E. (2026). Quantitation of Diastereomer Content in PS-Modified Synthetic Oligonucleotides Using cIMS-MS. Anal. Chem..

[93] Chatla K., Ayalew L., Yim M., Ko P., Lippold S., Hernandez G.I., Chua B.A., Patil D.P., Camperi J. (2026). Analytical Assessment of sgRNA Impurities and Their Impact on Functional Performance. Anal. Chem..

[94] Menneteau T., Addepalli B., Johnston T., Reidy C., Gorton M., Bouchenna J., Pittman N., Butré C.I., Mouvet D., Denbigh L. Advancing Gene Therapy: Enzyme Selection for Effective RNA Oligonucleotide Mapping|LCGC International. https://www.chromatographyonline.com/view/advancing-gene-therapy-enzyme-selection-for-effective-rna-oligonucleotide-mapping.

[95] Abernathy S., Rayhan A., Limbach P.A. (2024). Stationary Phase Effects in Hydrophilic Interaction Liquid Chromatographic Separation of Oligonucleotides. Analyst.

[96] Lobue P.A., Jora M., Addepalli B., Limbach P.A. (2019). Oligonucleotide Analysis by Hydrophilic Interaction Liquid Chromatography-Mass Spectrometry in the Absence of Ion-Pair Reagents. J. Chromatogr. A.

[97] Camperi J., Chatla K., Freund E., Galan C., Lippold S., Guilbaud A. (2025). Current Analytical Strategies for mRNA-Based Therapeutics. Molecules.

[98] Jiang T., Yu N., Kim J., Murgo J.-R., Kissai M., Ravichandran K., Miracco E.J., Presnyak V., Hua S. (2019). Oligonucleotide Sequence Mapping of Large Therapeutic mRNAs via Parallel Ribonuclease Digestions and LC-MS/MS. Anal. Chem..

[99] Beverly M., Hagen C., Slack O. (2018). Poly A Tail Length Analysis of in Vitro Transcribed mRNA by LC-MS. Anal. Bioanal. Chem..

[100] Maurer J., Vanluchene H., Tsalmpouris A., Morreel K., Camperi J., Sandra K., Guillarme D. (2025). Exploring the Potential of Oligonucleotide Mapping with Liquid Chromatography—Mass Spectrometry to Study the Primary Structure of mRNA. TrAC Trends Anal. Chem..

[101] Webb A.L.J., Welbourne E.N., Evans C.A., Dickman M.J. (2025). Characterisation and Analysis of mRNA Critical Quality Attributes Using Liquid Chromatography Based Methods. J. Chromatogr. A.

[102] Camperi J., Freund E., Galan C., Guilbaud A. (2025). Establishing Analytical Methods for RNA-Based Therapeutics Characterization. Characterizing Biotherapeutics.

[103] Kenderdine T., Nemati R., Baker A., Palmer M., Ujma J., FitzGibbon M., Deng L., Royzen M., Langridge J., Fabris D. (2020). High-Resolution Ion Mobility Spectrometry-Mass Spectrometry of Isomeric/Isobaric Ribonucleotide Variants. J. Mass Spectrom..

[104] Camperi J., Lippold S., Ayalew L., Roper B., Shao S., Freund E., Nissenbaum A., Galan C., Cao Q., Yang F. (2024). Comprehensive Impurity Profiling of mRNA: Evaluating Current Technologies and Advanced Analytical Techniques. Anal. Chem..

[105] Camperi J., Roper B., Freund E., Leylek R., Nissenbaum A., Galan C., Caothien R., Hu Z., Ko P., Lee A. (2024). Exploring the Impact of In Vitro-Transcribed mRNA Impurities on Cellular Responses. Anal. Chem..

[106] Campuzano I.D.G., Robinson J.H., Hui J.O., Shi S.D.-H., Netirojjanakul C., Nshanian M., Egea P.F., Lippens J.L., Bagal D., Loo J.A. (2019). Native and Denaturing MS Protein Deconvolution for Biopharma: Monoclonal Antibodies and Antibody–Drug Conjugates to Polydisperse Membrane Proteins and Beyond. Anal. Chem..

[107] Girgis M., Saed A., Alishetty S., Carrasco M., O’neill D., Baby N., Petruncio G., Hoemann C., Albano M., Peng X. (2026). Gauging Lipid Vulnerabilities in mRNA-LNPs under Various Environmental Stressors through TIMS-TOF Analysis. RSC Adv..

[108] Camperi J., Console G., Zheng L., Stephens N., Montti M., Roper B., Zheng M., Moshref M., Dagdas Y., Holder P. (2023). Comprehensive UHPLC- and CE-Based Methods for Engineered Cas9 Characterization. Talanta.

[109] Rogstad S., Yan H., Wang X., Powers D., Brorson K., Damdinsuren B., Lee S. (2019). Multi-Attribute Method for Quality Control of Therapeutic Proteins. Anal. Chem..

[110] Zhdanova P.V., Chernonosov A.A., Prokhorova D.V., Stepanov G.A., Kanazhevskaya L.Y., Koval V.V. (2022). Probing the Dynamics of Streptococcus Pyogenes Cas9 Endonuclease Bound to the sgRNA Complex Using Hydrogen-Deuterium Exchange Mass Spectrometry. Int. J. Mol. Sci..

[111] Kovalev M.A., Davletshin A.I., Karpov D.S. (2024). Engineering Cas9: Next Generation of Genomic Editors. Appl. Microbiol. Biotechnol..

[112] Camperi J., Moshref M., Dai L., Lee H.Y. (2022). Physicochemical and Functional Characterization of Differential CRISPR-Cas9 Ribonucleoprotein Complexes. Anal. Chem..

[113] Leney A.C., Heck A.J.R. (2017). Native Mass Spectrometry: What Is in the Name?. J. Am. Soc. Mass Spectrom..

[114] Lanucara F., Holman S.W., Gray C.J., Eyers C.E. (2014). The Power of Ion Mobility-Mass Spectrometry for Structural Characterization and the Study of Conformational Dynamics. Nat. Chem..

[115] O’Reilly F.J., Rappsilber J. (2018). Cross-Linking Mass Spectrometry: Methods and Applications in Structural, Molecular and Systems Biology. Nat. Struct. Mol. Biol..

[116] Shy B.R., Vykunta V.S., Ha A., Talbot A., Roth T.L., Nguyen D.N., Pfeifer W.G., Chen Y.Y., Blaeschke F., Shifrut E. (2023). High-Yield Genome Engineering in Primary Cells Using a Hybrid ssDNA Repair Template and Small-Molecule Cocktails. Nat. Biotechnol..

[117] Miller L.M., Hawkins L., Jarrold M.F. (2024). Compaction, Relaxation, and Linearization of Megadalton-Sized DNA Plasmids: DNA Structures Probed by CD-MS. J. Am. Soc. Mass Spectrom..

[118] Pearce E.L., Poffenberger M.C., Chang C.-H., Jones R.G. (2013). Fueling Immunity: Insights into Metabolism and Lymphocyte Function. Science.

[119] Repligen Corporation PATsmart^TM^ MAVEN® Online Glucose and Lactate Monitoring. https://www.repligen.com/maven.

[120] 908 Devices 908 Devices Launches the Rebel, the First At-Line Spent Cell Media Analyzer for Bioprocess Labs. https://908devices.com/news/908-devices-launches-the-rebel-the-first-at-line-spent-cell-media-analyzer-for-bioprocess-labs/.

[121] Repligen Corporation PATsmart REBEL XT At-Line Media Analyzer. https://www.repligen.com/rebelxt.

[122] Culberson A.L., Bowles-Welch A.C., Wang B., Kottke P.A., Jimenez A.C., Roy K., Fedorov A.G. (2023). Early Detection and Metabolic Pathway Identification of T Cell Activation by In-Process Intracellular Mass Spectrometry. Cytotherapy.

[123] Keshishian H., Addona T., Burgess M., Kuhn E., Carr S.A. (2007). Quantitative, Multiplexed Assays for Low Abundance Proteins in Plasma by Targeted Mass Spectrometry and Stable Isotope Dilution. Mol. Cell. Proteom..

[124] Meissner F., Scheltema R.A., Mollenkopf H.-J., Mann M. (2013). Direct Proteomic Quantification of the Secretome of Activated Immune Cells. Science.

[125] Nyman T.A., Lorey M.B., Cypryk W., Matikainen S. (2017). Mass Spectrometry-Based Proteomic Exploration of the Human Immune System: Focus on the Inflammasome, Global Protein Secretion, and T Cells. Expert Rev. Proteom..

[126] Byrnes J.R., Weeks A.M., Shifrut E., Carnevale J., Kirkemo L., Ashworth A., Marson A., Wells J.A. (2022). Hypoxia Is a Dominant Remodeler of the Effector T Cell Surface Proteome Relative to Activation and Regulatory T Cell Suppression. Mol. Cell. Proteom..

[127] European Medicines Agency Questions and Answers on Comparability Considerations for Advanced Therapy Medicinal Products (ATMP)—Scientific Guideline|European Medicines Agency (EMA). https://www.ema.europa.eu/en/questions-and-answers-comparability-considerations-advanced-therapy-medicinal-products-atmp-scientific-guideline.

[128] Lombard-Banek C., Pohl K.I., Kwee E.J., Elliott J.T., Schiel J.E. (2022). A Sensitive and Controlled Data-Independent Acquisition Method for Proteomic Analysis of Cell Therapies. J. Proteome Res..

[129] Ramello M.C., Benzaïd I., Kuenzi B.M., Lienlaf-Moreno M., Kandell W.M., Santiago D.N., Pabón-Saldaña M., Darville L., Fang B., Rix U. (2019). An Immunoproteomic Approach to Characterize the CAR Interactome and Signalosome. Sci. Signal..

[130] Salter A.I., Ivey R.G., Kennedy J.J., Voillet V., Rajan A., Alderman E.J., Voytovich U.J., Lin C., Sommermeyer D., Liu L. (2018). Phosphoproteomic Analysis of Chimeric Antigen Receptor Signaling Reveals Kinetic and Quantitative Differences That Affect Cell Function. Sci. Signal..

[131] Dakup P.P., Feng S., Shi T., Jacobs J.M., Wiley H.S., Qian W.-J. (2023). Targeted Quantification of Protein Phosphorylation and Its Contributions towards Mathematical Modeling of Signaling Pathways. Molecules.

[132] Louie R.H.Y., Cai C., Samir J., Singh M., Deveson I.W., Ferguson J.M., Amos T.G., McGuire H.M., Gowrishankar K., Adikari T. (2023). CAR+ and CAR− T Cells Share a Differentiation Trajectory into an NK-like Subset after CD19 CAR T Cell Infusion in Patients with B Cell Malignancies. Nat. Commun..

[133] Bai Z., Feng B., McClory S.E., de Oliveira B.C., Diorio C., Gregoire C., Tao B., Yang L., Zhao Z., Peng L. (2024). Single-Cell CAR T Atlas Reveals Type 2 Function in 8-Year Leukaemia Remission. Nature.

[134] Goldberg L., Haas E.R., Wu J., Garcia B., Urak R., Vyas V., Espinosa R., Munoz T., Bierkatz S., Pathak K.V. (2026). Immunometabolic Determinants of Long-Term Response in Leukemia Patients Receiving CD19 CAR T Cell Therapy. Nat. Commun..

[135] Hanley P.J. (2019). Fresh versus Frozen: Effects of Cryopreservation on CAR T Cells. Mol. Ther..

[136] Baust J.M., Campbell L.H., Harbell J.W. (2017). Best Practices for Cryopreserving, Thawing, Recovering, and Assessing Cells. Vitr. Cell. Dev. Biol..

[137] Pilely K., Johansen M.R., Lund R.R., Kofoed T., Jørgensen T.K., Skriver L., Mørtz E. (2022). Monitoring Process-Related Impurities in Biologics–Host Cell Protein Analysis. Anal. Bioanal. Chem..

[138] Rogers R.S., Nightlinger N.S., Livingston B., Campbell P., Bailey R., Balland A. (2015). Development of a Quantitative Mass Spectrometry Multi-Attribute Method for Characterization, Quality Control Testing and Disposition of Biologics. mAbs.

[139] Rogers R.S., Abernathy M., Richardson D.D., Rouse J.C., Sperry J.B., Swann P., Wypych J., Yu C., Zang L., Deshpande R. (2017). A View on the Importance of “Multi-Attribute Method” for Measuring Purity of Biopharmaceuticals and Improving Overall Control Strategy. AAPS J..

[140] Wang J., Wang C., Han X. (2019). Tutorial on Lipidomics. Anal. Chim. Acta.

[141] Köfeler H.C., Ahrends R., Baker E.S., Ekroos K., Han X., Hoffmann N., Holčapek M., Wenk M.R., Liebisch G. (2021). Recommendations for Good Practice in MS-Based Lipidomics. J. Lipid Res..

[142] Teachey D.T., Lacey S.F., Shaw P.A., Melenhorst J.J., Maude S.L., Frey N., Pequignot E., Gonzalez V.E., Chen F., Finklestein J. (2016). Identification of Predictive Biomarkers for Cytokine Release Syndrome after Chimeric Antigen Receptor T-Cell Therapy for Acute Lymphoblastic Leukemia. Cancer Discov..

[143] Hay K.A., Hanafi L.-A., Li D., Gust J., Liles W.C., Wurfel M.M., López J.A., Chen J., Chung D., Harju-Baker S. (2017). Kinetics and Biomarkers of Severe Cytokine Release Syndrome after CD19 Chimeric Antigen Receptor–Modified T-Cell Therapy. Blood.

[144] Gust J., Hay K.A., Hanafi L.-A., Li D., Myerson D., Gonzalez-Cuyar L.F., Yeung C., Liles W.C., Wurfel M., Lopez J.A. (2017). Endothelial Activation and Blood–Brain Barrier Disruption in Neurotoxicity after Adoptive Immunotherapy with CD19 CAR-T Cells. Cancer Discov..

[145] Santomasso B.D., Park J.H., Salloum D., Riviere I., Flynn J., Mead E., Halton E., Wang X., Senechal B., Purdon T. (2018). Clinical and Biological Correlates of Neurotoxicity Associated with CAR T-Cell Therapy in Patients with B-Cell Acute Lymphoblastic Leukemia. Cancer Discov..

[146] Anderson N.L., Anderson N.G., Haines L.R., Hardie D.B., Olafson R.W., Pearson T.W. (2004). Mass Spectrometric Quantitation of Peptides and Proteins Using Stable Isotope Standards and Capture by Anti-Peptide Antibodies (SISCAPA). J. Proteome Res..

[147] Picotti P., Aebersold R. (2012). Selected Reaction Monitoring–Based Proteomics: Workflows, Potential, Pitfalls and Future Directions. Nat. Methods.

[148] Daniels J.R., Cao Z., Maisha M., Schnackenberg L.K., Sun J., Pence L., Schmitt T.C., Kamlage B., Rogstad S., Beger R.D. (2019). Stability of the Human Plasma Proteome to Pre-Analytical Variability as Assessed by an Aptamer-Based Approach. J. Proteome Res..

[149] FDA-NIH Biomarker Working Group (2016). BEST (Biomarkers, EndpointS, and Other Tools) Resource.

[150] U.S. Food and Drug Administration M10 Bioanalytical Method Validation and Study Sample Analysis. https://www.fda.gov/regulatory-information/search-fda-guidance-documents/m10-bioanalytical-method-validation-and-study-sample-analysis.

